# Peptidomics analysis reveals changes in small urinary peptides in patients with interstitial cystitis/bladder pain syndrome

**DOI:** 10.1038/s41598-022-12197-2

**Published:** 2022-05-18

**Authors:** Md Shadman Ridwan Abid, Haowen Qiu, Bridget A. Tripp, Aline de Lima Leite, Heidi E. Roth, Jiri Adamec, Robert Powers, James W. Checco

**Affiliations:** 1 Department of Chemistry, University of Nebraska-Lincoln, Lincoln, NE USA; 2 Center for Biotechnology, University of Nebraska-Lincoln, Lincoln, NE USA; 3 The Nebraska Center for Integrated Biomolecular Communication (NCIBC), University of Nebraska-Lincoln, Lincoln, NE USA; 4 Department of Biochemistry, University of Nebraska-Lincoln, Lincoln, NE USA; 5 Redox Biology Center, University of Nebraska-Lincoln, Lincoln, NE USA

**Keywords:** Peptides, Proteomics, Biomarkers, Urology, Mass spectrometry

## Abstract

Interstitial cystitis/bladder pain syndrome (IC/BPS) is a chronic and debilitating pain disorder of the bladder and urinary tract with poorly understood etiology. A definitive diagnosis of IC/BPS can be challenging because many symptoms are shared with other urological disorders. An analysis of urine presents an attractive and non-invasive resource for monitoring and diagnosing IC/BPS. The antiproliferative factor (APF) peptide has been previously identified in the urine of IC/BPS patients and is a proposed biomarker for the disorder. Nevertheless, other small urinary peptides have remained uninvestigated in IC/BPS primarily because protein biomarker discovery efforts employ protocols that remove small endogenous peptides. The purpose of this study is to investigate the profile of endogenous peptides in IC/BPS patient urine, with the goal of identifying putative peptide biomarkers. Here, a non-targeted peptidomics analysis of urine samples collected from IC/BPS patients were compared to urine samples from asymptomatic controls. Our results show a general increase in the abundance of urinary peptides in IC/BPS patients, which is consistent with an increase in inflammation and protease activity characteristic of this disorder. In total, 71 peptides generated from 39 different proteins were found to be significantly altered in IC/BPS. Five urinary peptides with high variable importance in projection (VIP) coefficients were found to reliably differentiate IC/BPS from healthy controls by receiver operating characteristic (ROC) analysis. In parallel, we also developed a targeted multiple reaction monitoring method to quantify the relative abundance of the APF peptide from patient urine samples. Although the APF peptide was found in moderately higher abundance in IC/BPS relative to control urine, our results show that the APF peptide was inconsistently present in urine, suggesting that its utility as a sole biomarker of IC/BPS may be limited. Overall, our results revealed new insights into the profile of urinary peptides in IC/BPS that will aid in future biomarker discovery and validation efforts.

## Introduction

Interstitial cystitis/bladder pain syndrome (IC/BPS) is a chronic disorder characterized by pain, unpleasant pressure, or discomfort in the lower pelvis area^1–5^. IC/BPS can negatively impact a patient’s quality of life by affecting physical and mental well-being, interpersonal relationships, ability to work, and through the cost of medical care^6–8^. IC/BPS is diagnosed predominantly in women (45/100,000) over men (8/100,000)^2^, although there is evidence that IC/BPS may be underdiagnosed in both genders^9,10^. The etiology of IC/BPS is still not well understood, and multiple factors have been associated with IC/BPS pathophysiology. These factors include a thinning of the bladder epithelium, an increase in epithelial permeability, chronic inflammation, activation of an immune response, elevation of antiproliferation factors, and other conditions^1–5,11^. Symptomatic criteria to aid in the diagnosis of IC/BPS include bladder pain, an increase in urinary urgency, or the presence of glomerulations (pinpoint bleeding) and/or Hunner’s ulcers on the bladder wall. However, due to the high overlap of IC/BPS symptoms with other urological conditions, the diagnosis of IC/BPS remains a significant challenge and is usually based on eliminating other disorders. The methods traditionally used to aid in the diagnosis of IC/BPS are often highly invasive, which include hydrodistension, bladder biopsy, or cystoscopy^1,4^. IC/BPS has been identified as a priority research area by the American Urological Association (AUA). The AUA National Urology Research Agenda has emphasized understanding the mechanisms of IC/BPS and the development of non-invasive methods of diagnosis as major priorities^12,13^.

Identifying the molecular changes associated with IC/BPS and validating biomarkers represent important goals to aid in the understanding and diagnosis of this disorder^3,4,14,15^. Urine is attractive as a diagnostic source because it is easily accessible, its collection is non-invasive, and there are well-established protocols for the handling and storage of clinical samples^15,16^. Furthermore, urine may be particularly well suited as a source of biomarkers for IC/BPS because the pathology of this disorder commonly presents as changes in bladder physiology. Several prior studies have proposed putative IC/BPS biomarkers from urine using metabolomics^17–20^, proteomics^21–23^, or bioactivity experiments^24–27^. One promising putative biomarker, termed “antiproliferative factor” (APF), was identified based on the finding that urine and bladder cell releasates from IC/BPS patients had antiproliferative activity in cell-based activity assays^24–26^. Chromatographic purification of a bioactive component of urine/bladder tissue followed by mass spectrometry revealed a glycosylated nonapeptide (“APF peptide”), which appears to be a fragment of the frizzled-8 (FZD8) G protein-coupled receptor^27^. Subsequent studies confirmed that the APF peptide displays antiproliferative properties^27,28^, and appears to exert its effects through the CKAP4 receptor^29,30^. Despite convincing evidence that the antiproliferative bioactivity of urine may be used to discriminate IC/BPS patients^25^, to our knowledge no study has directly examined the urinary abundance of the APF peptide in IC/BPS patients versus healthy controls.

The promise of the APF peptide as a potential IC/BPS diagnostic suggests that small peptides (i.e., molecular weight < 10 kDa) may prove to be a rich source of urinary IC/BPS biomarkers. Small peptides can provide valuable information on the protease environment and disease state because most urinary peptides are proteolytic products of larger proteins^31–34^. In addition, peptides are relatively stable in urine compared to other biofluids (e.g., serum). However, aside from the APF peptide, small peptides have rarely been examined in IC/BPS. Traditional proteomics approaches incorporate enzymatic digestion, and also often include separation based on molecular weight, both of which preclude the identification of small endogenous peptides present in the sample^21–23^. In contrast, liquid chromatography-mass spectrometry (LC–MS)-based peptidomics studies specifically enrich and analyze small endogenous peptides and contain no enzymatic digestion steps^35–38^. Although some evidence has suggested that small- to medium-sized peptides may be used to differentiate IC/BPS urine from healthy controls^39^, the identity of these peptides remain unknown.

**Figure 1:**
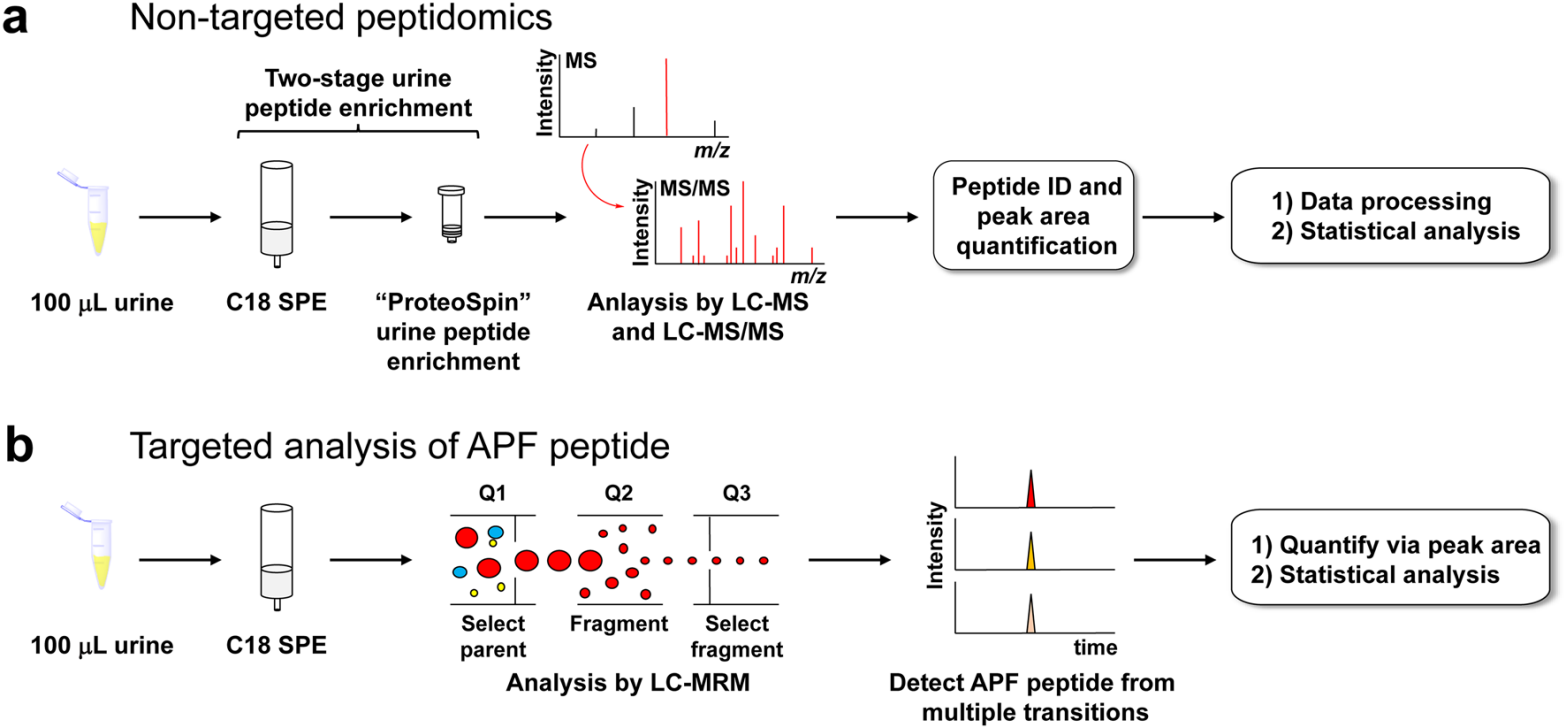
Workflows utilized in this study for the analysis of IC/BPS patient and healthy control urine. (**a**) Non-targeted LC–MS and LC–MS/MS peptidomics analysis used to identify urinary peptides that differ between IC/BPS patients and healthy controls. (**b**) Targeted LC-MRM analysis used to determine the relative quantities of the APF peptide in urine from both IC/BPS patients and healthy controls.

Here, we applied a non-targeted LC–MS and LC–MS/MS-based peptidomics approach to urine collected from IC/BPS patients and asymptomatic controls to explore differences in the profile of small urinary peptides in this disorder (Fig. 1a). These experiments identified several peptides that can be used to differentiate IC/BPS urine from controls. In parallel, we also developed and applied a targeted LC-multiple reaction monitoring (MRM) method to compare the relative quantities of the APF peptide present in urine from both IC/BPS patients and asymptomatic controls (Fig. 1b). Overall, our results reveal critical information on the urinary peptidome of IC/BPS patients. The identified peptides may inform future efforts to understand the molecular mechanisms of IC/BPS or may be used as the starting point for novel diagnostic approaches.

## Materials and methods

### General

500 mg C18 cartridges for solid phase extraction (SPE) were purchased from Thermo Fisher Scientific (product #60108-304). ProteoSpin Urine Protein Concentration Micro Kit for peptide enrichment was purchased from Norgen Biotek (product #17400). Micro BCA protein assay kit was purchased from Thermo Fisher Scientific (product #23235). LC–MS-grade solvents were used for all sample preparation prior to LC–MS analysis. Synthetic APF peptide was purchased from Vivitide (product #CAR-24007-v). Unless otherwise specified, all other reagents and solvents were purchased from Thermo Fisher Scientific or Millipore Sigma.

### Patient urine sample information

Urine samples from IC/BPS patients and healthy controls were obtained from the National Institute of Diabetes and Digestive and Kidney Diseases (NIDDK) Central Repository’s Multidisciplinary Approach to the Study of Chronic Pelvic Pain (MAPP) Research Network^40,41^. The University of Nebraska-Lincoln Institutional Review Board (IRB) reviewed the secondary use of materials and data obtained from the study available here: https://repository.niddk.nih.gov/studies/mapp_ep/. The University of Nebraska-Lincoln IRB determined that the study was exempt from further IRB review. All analysis and ethical storage of data and specimens adhered to the University policies associated with Responsible Conduct of Research and the IRB. All subjects provided informed consent for long-term storage and sharing of biospecimens and data per the original observational cohort study protocol.

Samples were categorized as IC/BPS if they had a ≥ 22 total Genitourinary Pain Index (GUPI) score^42^. Age-matched control samples were from individuals with < 22 total GUPI score. More information on patient symptom scores and demographics can be found in Table S1. Upon receipt, samples were stored at − 80 °C until analysis.

### Sample preparation for non-targeted peptidomics

To account for batch effects, urine samples were randomly distributed into sample sets for processing and injection, where each sample set contained an equal number of IC/BPS and control samples. Sample distribution into sample sets was predefined before the start of sample processing.

Patient or control urine samples (100 μL) were acidified with 1 μL of 10% formic acid in water (0.1% formic acid final concentration) and desalted via SPE with 500 mg C18 cartridges. For each sample, the SPE column was activated with 50% acetonitrile/water and 0.1% formic acid (2 × 500 μL) and then equilibrated with 3% acetonitrile/water and 0.1% formic acid (2 × 500 μL). Acidified urine samples were then loaded onto the column, followed by washes of 3% acetonitrile/water and 0.1% formic acid (2 × 500 μL). Finally, bound peptides were eluted with consecutive washes of 25% acetonitrile/water and 0.1% formic acid (500 μL), 50% acetonitrile/water and 0.1% formic acid (500 μL), and 75% acetonitrile/water and 0.1% formic acid (500 μL). Eluted samples were dried in a vacuum concentrator and redissolved in 20 μL of 100% water and 0.1% formic acid. Estimated peptide concentrations were determined by a bicinchoninic acid (BCA) assay. A ProteoSpin Urine Protein Concentration Micro Kit was used to further enrich for urine peptides, which included a binding buffer, a wash solution, an elution solution, and protein neutralizer^34^. Each urine sample was diluted with 100 μL water and 0.1% formic acid and 4 μL of binding buffer. A pH of 3–4 for each sample was verified with pH paper. The column was washed twice by adding 500 μL of wash solution and centrifuging at 3300×*g* for 2 min. The sample was loaded onto the column and centrifuged at 3300×*g* for 2 min. The column was then washed twice by adding 500 μL of wash solution and centrifuging at 3300×*g* for 2 min. Finally, peptides were eluted with 100 μL of elution solution by centrifuging at 3300×*g* for 2 min into clean collection tubes containing 9.3 μL of protein neutralizer. The sample was then dried and stored at − 20 °C until LC–MS analysis.

### LC–MS analysis for non-targeted peptidomics

Dried peptide extracts were dissolved in 9 μL of water and 0.1% formic acid. For each prepared peptide extract, 5 μL was injected onto a Waters ACQUITY UPLC M-Class system coupled to a Waters Xevo G2-XS quadrupole time-of-flight (Q-ToF) mass spectrometer equipped with a nano-electrospray ionization source (Waters Z spray NanoLockSpray). For LC, the mobile phase was composed of water and 0.1% formic acid (Solvent A) and acetonitrile and 0.1% formic acid (Solvent B). Peptide extracts were first loaded onto a Waters nanoEase M/Z symmetry C18 trap column (180 μm × 20 mm, product #186008821) for on-line desalting prior to separation, with a loading solvent of 1% B and a flow rate of 5 μL/min for 10 min. Peptide separations were performed on Waters nanoEase-C18 column (75 μm × 250 mm, product# 186008818) with a flow rate of 0.35 μL/min. The column compartment was set to 35 °C and the autosampler compartment was maintained at 8 °C. At the time of injection, the solvent mixture was held at 3% B. A 40-min gradient from 3 to 40% B was initially applied, followed by a 4-min gradient to increase B to 85%, and then a 4-min washing step at 85% B. The column was re-equilibrated to 3% B with a total run time of 60 min. MS and MS/MS (collision-induced dissociation fragmentation) analysis was performed in positive ion mode over a mass range of 100–2000 *m/z*. Precursor ions were selected for MS/MS using data-dependent acquisition with 3 precursor ions selected for each MS scan based on a peak intensity threshold of 5000. The MS scan time was 0.5 s, and the MS/MS scan time was 1 s.

The LC–MS data were processed using PEAKS Studio X Pro software (Bioinfomatics Solutions Inc.)^43,44^. The Human SwissProt reference proteome from UniProt^45^ was used for peptide identifications. The parent mass error tolerance was set to 10 ppm and the fragment mass error tolerance was set to 0.1 Da. The false discovery rate (FDR) threshold was set to 1%. PEAKS Q Module for label-free quantification was used to calculate peptide peak areas and to reduce false missing values via ID-transfer. The Q module mass error tolerance was set to 20.0 ppm and the retention time shift tolerance was set to 2.5 min. All identified peptides and peak areas (sum of peak areas for each charge state) were exported from the PEAKS Q Module without normalization. For statistical analysis, data were preprocessed by considering peptide features present in at least 50% of the samples with unambiguous identifications. Imputation was performed using the k-nearest neighbors algorithm (knn; k = 7)^46^, and normalized using EigenMS^47,48^. Data preprocessing was prepared using the R statistical software version 4.0.3^49^. Differential features were assessed using parametric (*t* test) and non-parametric (Wilcoxon rank sum) statistical tests to account for the degree, direction, and rank of differences between the patient and control groups. For both statistical tests, the Benjamini–Hochberg (BH) procedure was applied to correct for multiple hypothesis testing^50^. Peptides were considered differential with a BH corrected p-value of less than 0.05 in both statistical tests. For the purposes of data plotting, reported p-value is termed “p_adj_” and is the minimum of the BH-corrected p-value from the *t* test and the Wilcoxon rank sum test. The fold change (FC) was calculated for each peptide to quantify a magnitude difference between sample groups, with a positive FC indicating higher abundance in the IC/BPS group. Multivariate analysis, principal component analysis (PCA) and partial least squares-discriminant analysis (PLS-DA), were performed using R packages pcaMethods and ropls, respectively^49–53^. The five peptides with variable importance in projection (VIP) coefficients greater than or equal to 2.00 were used to build logistic regression and random forest classification models, and the performance of the models were evaluated by calculating the area under the curve (AUC) values^54^.

### Targeted analysis of APF

To develop an MRM method, the highest single charged (1482.8 *m*/*z*) and double charged (741.88 *m*/*z*) states of the APF peptide were used as precursor ions, and the five product ions (539.32 *m*/*z*, 620.38 *m*/*z*, 626.39 *m*/*z*, 638.39 *m*/*z*, 826.5 *m*/*z*) were used as transitions to monitor the APF peptide. Based on these results, the 1482.8 *m*/*z* precursor ion and the 826.5 *m*/*z* transition ion were chosen for APF peptide quantification. The FMRGF-NH_2_ peptide was used as an internal standard, which was synthesized using Fmoc solid-phase peptide synthesis. The highest single charged state for the precursor ion (656.34 *m*/*z*) and the five product ions (509.26 *m*/*z*, 492.23 *m*/*z*, 435.21 *m*/*z*, 279.11 *m*/*z*, 222.12 *m*/*z*) were used to monitor the internal standard. The 435.21 *m*/*z* product ion was chosen for quantification of the internal standard.

### Sample preparation for targeted APF quantification

Patient or control urine samples (100 μL) were acidified with 1 μL of 10% formic acid in water (0.1% formic acid final concentration) and desalted via SPE with 500 mg C18 cartridges, as described above. After SPE, eluted samples were dried in a vacuum concentrator and redissolved in 30 μL of 100% water and 0.1% formic acid. Estimated peptide concentrations were determined with a BCA assay.

### LC-MRM analysis

27 μL of each peptide extract was spiked with 3 μL of a 10 μM solution of FMRGF-NH_2_ for a final concentration of 1 μM for the internal standard. 5 μL of each sample was injected onto an Agilent 1260 Infinity HPLC system coupled to an Agilent 6410 triple quadrupole mass spectrometer. Peptide separations were performed on Eclipse Plus-C18 (2.1 × 50 mm) column. The flow rate was 100 μL/min, and the mobile phase was composed of water and 0.1% formic acid (Solvent A) and acetonitrile and 0.1% formic acid (Solvent B). At the time of injection, the solvent mixture was held at 5% B. An 18-min gradient from 5 to 35% B was initially applied, followed by a 7-min gradient that increased B to 100%, and then a 1-min washing step at 100% B. The column was re-equilibrated to 5% B for a total run time of 40 min. MS and MS/MS data acquisition was performed in the positive ion mode. For MS/MS analysis, 45 V collision energy was applied for both the APF peptide and the internal standard. For all MRM channels, the mass tolerance for the precursor and transition ions were set to 1 amu. To account for variability in sample injection, the APF peptide peak area was normalized by dividing by the peak area for the internal standard. An unpaired *t* test was performed to compare sample means for the samples in which the APF peptide was detected.

## Results

### Non-targeted LC–MS peptidomics

Human urine samples were obtained from the MAPP Research Network, an observational cohort study designed to investigate IC/BPS in women and men, and Chronic Prostatitis (CP)/Chronic Pelvic Pain Syndrome (CPPS) in men^40,41^. IC/BPS urine samples were from patients with a high combined pain, urine, and quality of life symptom GUPI scores of 31 ± 6 (Table S1)^42^, which indicated a relatively high severity of the disorder. Age-matched control samples were from non-IC/BPS individuals with low total GUPI scores of 2 ± 3 (Table S1). Urine samples were only obtained from female patients to reduce variability due to gender and because IC/BPS disproportionately affects women^2^.

A total of 43 IC/BPS and 44 control urine samples were prepared for non-targeted LC–MS and LC–MS/MS peptidomics analysis to examine changes in urinary peptide content associated with IC/BPS (Fig. 1a). Briefly, an equal volume (100 μL) of each patient’s urine sample was thawed and subjected to a two-stage extraction protocol previously found to be beneficial for the isolation and identification of small urinary peptides by LC–MS and LC–MS/MS^34^. The approach utilized sample enrichment by C18 SPE followed by further enrichment of urinary peptides using a commercially available ProteoSpin kit (Norgen Biotek Corp.). After the C18 SPE desalting step, a BCA assay performed on sample extracts indicated a modestly higher average apparent protein concentration (~ 1.4-fold) in IC/BPS samples relative to control samples (Fig. S1). Our preliminary studies indicated that the protein concentration after the second ProteoSpin enrichment step was below the limit of detection for a BCA assay, eliminating a reason to measure the apparent protein concentration after this second enrichment step. Instead, the entirety of the extracts from the SPE stage were carried forward to the ProteoSpin stage, after which equal volumes were injected for analysis by LC–MS and LC–MS/MS.

**Figure 2:**
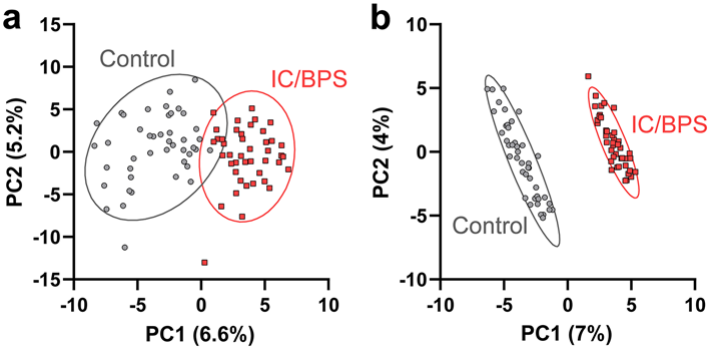
(**a**) PCA and (**b**) PLS-DA scores plots of non-targeted LC–MS and LC–MS/MS data after preprocessing and normalization by EigenMS shows separation between IC/BPS patients (red) and healthy controls (grey) based on peptide profiles.

A total of 995 individual peptides were identified (fragments from 149 different proteins) from the LC–MS and LC–MS/MS datasets using PEAKS Studio proteomics software^43,44^ with a 1% false discovery rate (FDR) threshold. Only 212 peptides with unambiguous sequence assignments were detected in at least 50% of the samples, a level appropriate for downstream univariate statistical analysis (Supporting Document 1). After imputation using knn (k = 7)^46^, the data was normalized using EigenMS^47,48^ to account for sample-to-sample variability (Fig. S2). Scores plots from both PCA and PLS-DA models showed a clear separation between the IC/BPS and control groups (Fig. 2).

**Figure 3:**
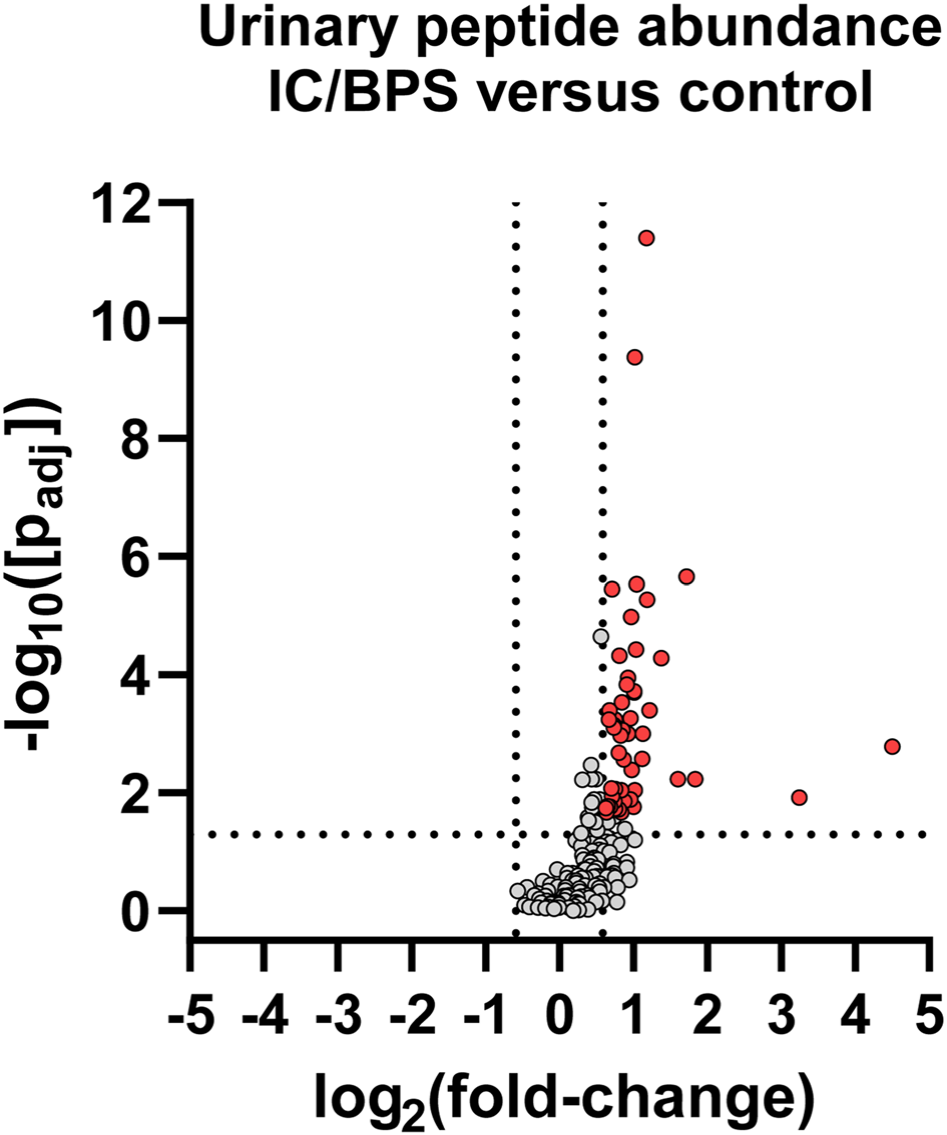
Volcano plot of detected peptides. Red data points indicate the significantly changing peptides with a > 1.5-fold higher abundance in the urine samples from the IC/BPS patients. The two vertical lines are demarcation points for the 1.5-fold up and down changes between the groups. The horizontal line marks a corrected p-value of 0.05. These data show that most identified peptides are of higher abundance in the IC/BPS patients.

A univariate analysis identified 71 peptides with a p-value less than 0.05 from both the BH-corrected *t* test and Wilcoxon rank-sum tests (Fig. S3, Supporting Document 1). These peptides exhibited a statistically significant differential abundance between IC/BPS and healthy controls. The 71 differential peptides were degradation products from a variety of proteins, including several previously associated with IC/BPS from prior proteomics studies (Table 1)^21–23^. Interestingly, all of the differential peptides were found in a higher abundance in IC/BPS relative to control samples (Fig. 3). The 18 peptides with the largest − log10[p_adj_] values are shown in Table 2, with linear fold-changes from 1.5 to 3.3 (see also Fig. S4).

**Table 1:** List of proteins that generated the 71 significant urinary peptides identified in this study, along with their Uniprot IDs, gene names, and the number of peptides detected from each protein. Proteins names marked with a * were previously identified as changing in IC/BPS by prior proteomic studies^21–23^.

Protein name	Protein ID (Uniprot)	Gene name	Number of peptides
Osteopontin*	OSTP	SPP1	9
Uromodulin*	UROM	UMOD	6
Polymeric immunoglobulin receptor	PIGR	PIGR	5
CD99 antigen	CD99	CD99	3
Inter-alpha-trypsin inhibitor heavy chain H4*	ITIH4	ITIH4	3
Secreted and transmembrane protein 1	SCTM1	SECTM1	3
Protein AMBP	AMBP	AMBP	2
Complement C1r subcomponent-like protein	C1RL	C1RL	2
Collagen alpha-1(III) chain	CO3A1	COL3A1	2
Endothelial protein C receptor	EPCR	PROCR	2
Hemoglobin subunit beta	HBB	HBB	2
Insulin	INS	INS	2
Kininogen-1*	KNG1	KNG1	2
Basement membrane-specific heparan sulfate proteoglycan core protein*	PGBM	HSPG2	2
Roundabout homolog 4	ROBO4	ROBO4	2
Alpha-1-acid glycoprotein 1/2*	A1AG1/A1AG2	ORM1/ORM2	1
Alpha-1-antitrypsin	A1AT	SERPINA1	1
Actin, cytoplasmic 1/2	ACTB/ACTG	ACTB/ACTG1	1
Albumin*	ALBU	ALB	1
Collagen alpha-1(I) chain	CO1A1	COL1A1	1
Collagen alpha-1(X) chain	COAA1	COL10A1	1
Collagen alpha-1(XVIII) chain	COIA1	COL18A1	1
Collagen alpha-1(XXII) chain	COMA1	COL22A1	1
Cystatin-A	CYTA	CSTA	1
Fibrinogen beta chain	FIBB	FGB	1
Gelsolin	GELS	GSN	1
Histone H1.2	H12	H1-2	1
Histone H1.4	H14	H1-4	1
Insulin-like growth factor-binding protein 7	IBP7	IGFBP7	1
Insulin-like growth factor II	IGF2	IGF2	1
Immunoglobulin heavy constant gamma 1/2	IGHG1/IGHG2	IGHG1/IGHG2	1
Kallikrein-1	KLK1	KLK1	1
Vesicular integral-membrane protein VIP36	LMAN2	LMAN2	1
Nidogen-1*	NID1	NID1	1
Neuropeptide W precursor	NPW	NPW	1
Phosphoinositide-3-kinase-interacting protein 1	P3IP1	PIK3IP1	1
Extracellular superoxide dismutase [Cu–Zn]	SODE	SOD3	1
Neurosecretory protein VGF	VGF	VGF	1
Xylosyltransferase 1	XYLT1	XYLT1	1

**Table 2:** List of peptides with − log10[p_adj_] ≥ 3.40, along with their Uniprot IDs, linear and log2 fold-change values (IC/BPS vs. control), − log10[p_adj_] value, and VIP score from PLS-DA.

Peptide sequence	Protein ID (Uniprot)	Fold-change (IC/BPS vs. control)		− log_10_[p_adj_]	VIP
		Lin	Log2		
DADLADGVSGGEGKGGSDGGGSHRKEGEEADAPGVIPGIVG	CD99	2.3	1.2	11.40	2.23
DGVSGGEGKGGSDGGGSHRKEGEEADAPGVIPGIVG	CD99	2.0	1.0	9.38	2.59
IPVKQADSGSSEEKQLYNKYPDAVA	OSTP	3.3	1.7	5.66	1.75
IPVKQADSGSSEEKQLYNKYPDAVAT	OSTP	2.1	1.0	5.54	1.96
DLADGVSGGEGKGGSDGGGSHRKEGEEADAPGVIPGIVG	CD99	1.6	0.7	5.45	2.13
YRITEATKTVGSDTF	KNG1	2.3	1.2	5.28	2.01
VSWVPPPAENHNGIIRG	ROBO4	2.0	1.0	4.98	2.00
VGGGEQPPPAPAPRRE	XYLT1	1.5	0.6	4.65	2.01
EDPQGDAAQKTDTSHHDQDHPTF	A1AT	2.1	1.0	4.43	1.91
EEKAVADTRDQADGSRASVDSGSSEEQGGSSRALVSTL	PIGR	1.8	0.8	4.33	1.77
SGSVIDQSRVLNLGPITR	UROM	2.6	1.4	4.29	1.88
IILEHHVAQEPSPGQPSTF	PGBM	1.9	0.9	3.95	1.71
DEELGGTPVQSRVVQGKEPAHL	GELS	1.9	0.9	3.84	1.75
DDQSAETHSHKQSRLY	OSTP	2.0	1.0	3.72	1.61
AASLAGPHSIVGRA	SODE	2.0	1.0	3.70	1.77
FAEEKAVADTRDQADGSRASVDSGSSEEQGGSSRALVSTL	PIGR	1.8	0.8	3.54	1.71
DGVPGKDGPRGPTGP	CO3A1	1.6	0.7	3.40	1.58
LAQELPQQLTSPGYPEPYGKGQESSTD	C1RL	2.3	1.2	3.40	1.65

**Figure 4:**
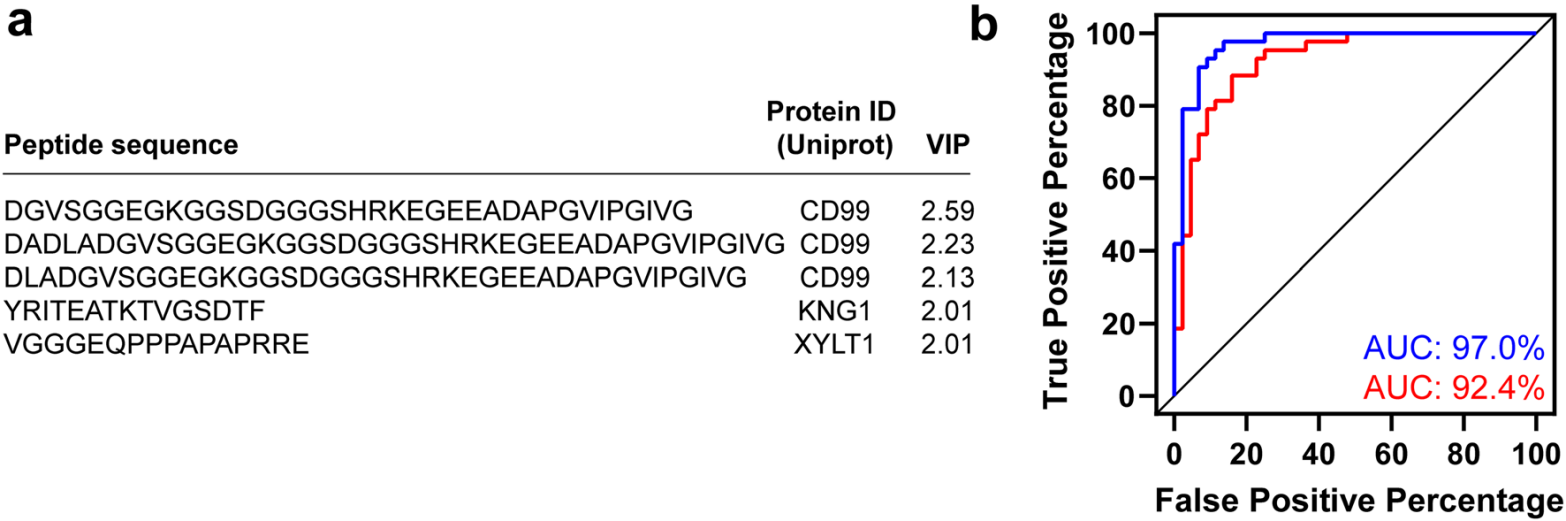
(**a**) List of five peptides with VIP scores > 2.00 from PLS-DA. These five peptides were used for further ROC analysis. (**b**) The ROC curves from the logistic regression (blue) or random forest (red) model using the five peptides listed in panel (**a**). AUC values indicate the area under the curve for each model.

PLS-DA identified five peptides with a VIP coefficient greater than 2.00 and a p-value less than 0.05 from both a BH-corrected *t* test and a Wilcoxon rank-sum test (Fig. 4a). These five peptides were used as input to calculate a ROC curve, which yielded an AUC of 97.0% from a logistic regression model or an AUC of 92.4% from a random forest model (Fig. 4b). These results indicate that the five peptides provide a high predictive capability to distinguish urine from IC/BPS patients and healthy controls. ROC curves using the individual peptides generally showed a reduced predictive capability (Fig. S5), demonstrating that the combination of peptide abundances is required for high predictive ability.

### Targeted analysis of APF peptide

Based on prior literature evidence suggesting that the APF peptide may be a biomarker for IC/BPS, we sought to compare the quantities of the APF peptide in urine from IC/BPS patients relative to urine from age-matched healthy controls. However, our non-targeted LC–MS and LC–MS/MS peptidomics datasets did not detect the APF peptide in the interrogated urine samples. This result suggests that either the APF peptide is present at concentrations below the limit of detection for our method, or the APF peptide was lost during our sample preparation steps. To address these possibilities, we aimed to develop a targeted method to quantify the relative abundances of the APF peptide in urine samples.

An LC-MRM method to quantify APF peptide abundance from urine peptide extracts was developed using a synthetic APF peptide standard and commercially available urine samples. Fragment ions used to develop the MRM channels were chosen based on experimentally determined MS/MS spectra for the synthetic APF peptide (Figs. S6 and S7). In control experiments, the APF peptide spiked into the standard urine samples was detectable by LC-MRM after a single stage of C18 SPE but was not detectable if the two-stage enrichment method was used (Fig. S8). This observation demonstrates the APF peptide was not effectively enriched during the second ProteoSpin enrichment step and explains why the APF peptide was not detected in our non-targeted peptidomics experiments. Thus, for APF peptide detection, we extracted peptides from urine samples using a single SPE stage followed by injection onto the LC–MS system in MRM mode.

**Figure 5:**
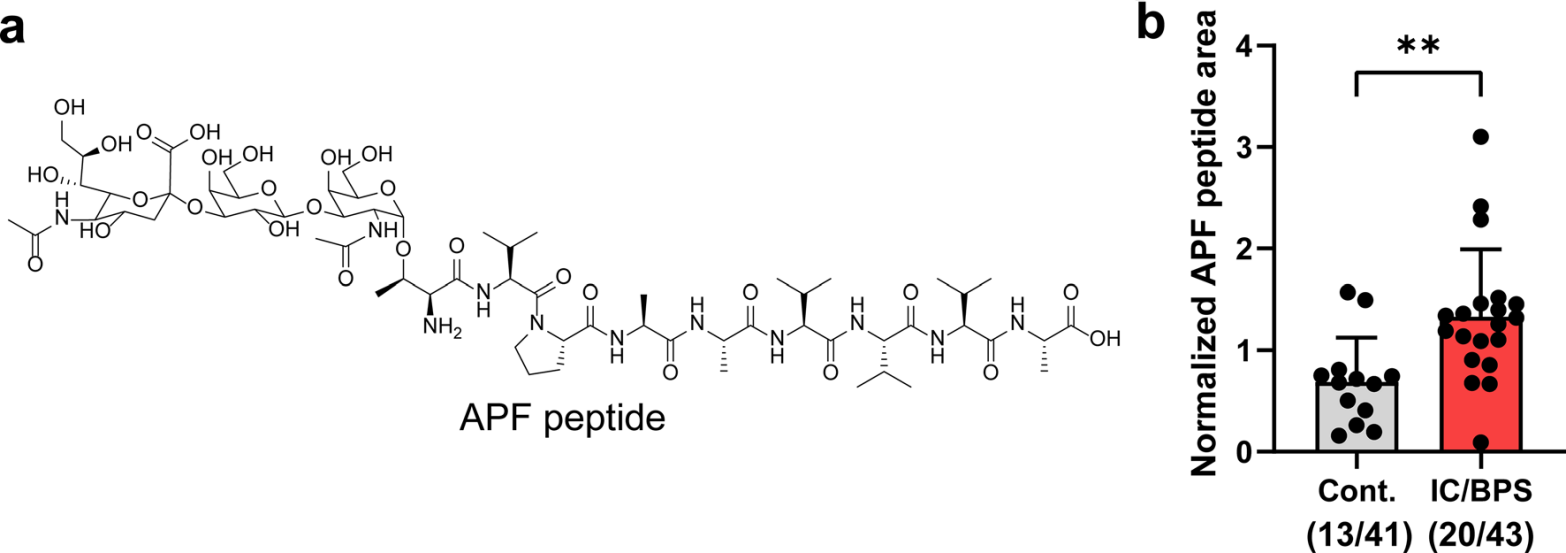
(**a**) Chemical structure of APF peptide. (**b**) Relative abundance of the APF peptide in the urine samples as assessed by LC-MRM. All data were normalized by dividing the peak area for the APF peptide by the peak area for the internal standard peptide (FMRGF-NH_2_). The APF peptide was detected in 20 IC/BPS patient samples and 13 control samples. The black points show the normalized peak areas for each sample. Bars show the mean and standard deviation of the normalized peak areas. **p < 0.01 (unpaired *t* test).

We analyzed 84 human urine samples from 43 IC/BPS patients and 41 healthy controls for the APF peptide. An equal volume (100 μL) of each urine sample was processed by C18 SPE, spiked with the standard peptide (FMRGF-NH_2_), and then analyzed with our LC-MRM protocol. From this procedure, we detected the APF peptide in 20 out of the 43 IC/BPS patient samples (Fig. 5 and Fig. S9). Surprisingly, we also detected the APF peptide in 13 out of the 41 control samples. For samples in which the APF peptide was detected, we observed an ~ 2-fold higher average APF peptide signal (p < 0.01) in the urine from IC/BPS patients relative to healthy controls (Fig. 5). The APF peptide was not detected in 23 of the IC/BPS samples or in 28 of the control samples. Overall, the presence or absence of the APF peptide did not correlate with GUPI score (Fig. S10a). Similarly, the relative abundance of the APF peptide did not correlate well with GUPI score (Fig. S10b). Notably, the detection of APF was more frequent in samples with a higher protein concentration after the SPE step, as determined by BCA assay (Fig. S11). This suggests that the abundance of APF peptide is primarily dependent on the total protein concentration in the urine and not just the presence or severity of IC/BPS.

## Discussion

Urine is an attractive source for diagnostic markers due to its easy accessibility and established procedures for non-invasive collection, handling, and storage. For IC/BPS, urine may be especially appropriate for examining molecular changes because the pathophysiology of IC/BPS includes a number of abnormalities in the bladder wall, which comes in direct contact with urine as it is stored in the bladder. Indeed, prior proteomics and metabolomics studies have identified changes in urinary protein and small molecule content in IC/BPS urine compared to controls^17–23^. The most promising IC/BPS biomarker thus far is the APF peptide, which was identified from cell-based assays showing decreased proliferative activity when exposed to IC/BPS patient urine or cellular releasates^24–27^. Inspired by this observation, we chose to investigate the presence of small urinary peptides associated with IC/BPS. To begin, we performed the first exploration into the IC/BPS urinary peptidome, with the goal of identifying small urinary peptides other than the APF peptide that may serve as diagnostic tools for IC/BPS. In parallel, we also compared the relative quantities of the APF peptide between IC/BPS patients and control samples to provide more insight into the utility of the APF peptide as an IC/BPS biomarker.

Our non-targeted peptidomics analysis of patient urine identified 71 peptides elevated in IC/BPS patients relative to controls. These peptides were proteolytic degradation products from 39 different proteins (Table 1). The general increase in the abundance of many small peptides in IC/BPS is consistent with prior studies showing increased protease abundance in urine/bladder cells associated with IC/BPS^23,55–58^. For example, prior studies have identified that urine from IC/BPS patients experiencing pain and low bladder capacity have a significantly increased abundance of active neutrophil elastase^23^, a serine protease with relatively broad substrate specificity^59^. The higher neutrophil elastase abundance and activity was determined to be responsible for the greatly increased abundance of albumin fragments in patient’s urine^23^. Consistent with these results, proteolytic fragments of albumin were also found in the urine of cats with feline idiopathic cystitis, a feline disorder with many similarities to IC/BPS^60^. Interestingly, this same study also found a general increase in protein abundance in urine from cats with feline idiopathic cystitis. As a result, our finding that small urinary peptides increased in abundance in IC/BPS urine is likely a result of increased proteolysis occurring in the urine or the bladder wall, which is consistent with the inflammatory and immune responses (e.g., mast cell activation) associated with this disorder^1–5,11^.

Eight of the 39 proteins found to generate statistically significant urinary peptides in our study have been previously implicated in IC/BPS in prior proteomics studies (Table 1, highlighted with *)^21–23^. Three proteins, osteopontin (OSTP), alpha-1-acid glycoprotein 1 (A1AG1), and inter-alpha-trypsin inhibitor heavy chain H4 (ITIH4), were previously found to be increased in the urine of IC/BPS patients relative to healthy controls^21,22^. Our peptidomics results were consistent with these prior studies, as peptides from these proteins were found to be in higher abundance in urine from IC/BPS patients. Notably, our study also revealed the specific sequences of the small peptides from the proteins enriched in the urine from IC/BPS patients. Our results also showed an increase in a degradation product from albumin, consistent with prior outcomes from human IC/BPS and feline idiopathic cystitis studies^23,60^. Prior proteomics studies also showed a decrease in proteins uromodulin (UROM), kininogen-1 (KNG1), basement membrane-specific heparan sulfate proteoglycan core protein (PGBM), and nidogen-1 (NID1) in the urine of IC/BPS patients relative to controls^21,22^. Conversely, our results showed an increase in peptides generated from these proteins in IC/BPS urine. These outcomes may not be contradictory since overall intact protein levels may decrease in concert with an increase in proteolytic fragments resulting from protein degradation. Our results highlight the ability of peptidomics to obtain information that was not accessible in prior proteomic efforts due to the inclusion of protease digestion in workflows, complementing standard proteomics methods.

In addition to detecting peptides from several proteins previously associated with IC/BPS, we also identified peptides from several proteins that have not been previously implicated in this disorder. For example, significant peptides were generated from CD99 antigen (CD99), polymeric immunoglobulin receptor (PIGR), xylosyltransferase 1 (XYLT1), alpha-1-anti-trypsin (A1AT), and gelsolin (GELS). Peptides from each of these proteins have previously been detected in prior urinary peptidomics experiments^32–34,61^, and our study shows an increase in specific peptides from these proteins in IC/BPS patient urine. It is not clear if these proteins themselves play a physiological role in IC/BPS, or if the general increase in these specific peptides is a consequence of other processes, such as increased protease degradation. Regardless, ROC analysis using several peptides in combination was found to reliably classify urine as IC/BPS or control (Fig. 5), suggesting that these peptides may be useful to inform future diagnostic efforts.

Parallel to our non-targeted peptidomics analysis, we also carried out a targeted analysis of the APF peptide using LC-MRM. Prior studies have demonstrated that an antiproliferative activity as assessed by cell-based proliferation assays can reliably differentiate IC/BPS urine from controls^25^. This antiproliferative activity has been attributed to the APF peptide^27^. The gene encoding the APF peptide was found to be consistently overexpressed in IC/BPS patient bladder epithelial cells samples relative to controls as determined by Northern blot^27^. Chavda et al. developed a surface plasmon resonance-based assay to detect a synthetic desialylated APF peptide analogue (as-APF) spiked into control urine, but this assay was not used to quantify the APF peptide from IC/BPS urine^30^. To our knowledge, our study is the first to directly measure the APF peptide from IC/BPS urine and to compare its abundance to control urine. Our LC-MRM assay showed that the APF peptide was only detectable in 47% of the IC/BPS urine samples analyzed, but it was also found in 32% of the control samples. For urine samples that did contain a detectable concentration of the APF peptide, the peptide was found to be in a higher abundance in the urine from IC/BPS patients compared to healthy controls (Fig. 5). These results suggest that although APF peptide may sometimes be moderately increased in IC/BPS patients, it may be difficult to rely on this peptide as a sole biomarker for IC/BPS due both to the inconsistency of this peptide’s presence in IC/BPS patient urine and its relatively high abundance in control urine.

The detectability of the APF peptide in urine was correlated with a higher overall apparent protein concentration after SPE (Fig. S11). This result suggests the abundance of APF peptide in a urine sample may be more dependent on the total protein concentration than IC/BPS diagnosis. Of course, IC/BPS may also have a modest impact on the total urine protein concentration (Fig. S1). Importantly, our LC-MRM method only targeted the full sequence of the APF peptide (Neu5Acα2-3Galβ1-3GalNAcα-O-TVPAAVVVA-OH, Fig. 5a)^27^. Other forms of the APF peptide (e.g., truncated or modified) would not have been detected because of the specificity of LC-MRM for target analytes based on parent and fragment masses. Thus, it is possible that alternative forms of the APF peptide may be present in IC/BPS patient samples, and these may prove to be more reliable as IC/BPS biomarkers than the form studied here. Indeed, prior studies have suggested that the desialylated as-APF peptide may occur naturally, and structure–activity studies found that APF peptide derivatives truncated at either the N- or C-terminus retain high biological activity^27,30,62,63^. Future studies may benefit from examining alternative forms of the APF peptide, or examining other molecules that may also be responsible for the antiproliferative activity found in IC/BPS urine.

In addition to analyzing small urinary peptides, we also aimed to characterize changes in urinary metabolites associated with IC/BPS. To accomplish this goal, we applied our previously developed LC–MS and NMR metabolomics pipelines to our MAPP patient urine samples^64–67^. However, after data collection and analysis, we were unable to find statistically significant differences in metabolites between the IC/BPS and control samples using a variety of univariate and multivariate statistical methods (see Supporting Information, Fig. S12). This result contrasts with prior studies that identified urine metabolite markers differentiating IC/BPS and control urine^17,19,20^.

It is unclear why we did not identify differences in the metabolite profiles, but it is plausible that the IC/BPS pathology simply does not significantly perturb the urinary metabolome. It is important to note that these prior studies each identified completely different urinary metabolites that distinguished IC/BPS from healthy controls. In one study, tyramine and 2-oxoglutarate were increased in the urine from IC/BPS patients^20^ while etiocholan-3α-ol-17-one sulfate was observed to increase in a second study^19^, and phenylacetylglutamine was observed to increase in a third study^17^. This lack of consistency across three distinct metabolomics studies raises doubts on the reliability of any of these metabolites to serve as a potential biomarker for IC/BPS. Further concerning was the observation by Parker et al.^19^ that the metabolic profile of the IC/BPS patients could be divided into two subgroups, where the metabolome from one IC/BPS subgroup was consistent with healthy controls. Thus, our observed lack of a distinct metabolic signal in the urine from IC/BPS is at least partially consistent with this prior result. Notably, Parker et al.^19^ also received their urine samples from the MAPP Research Network, but completed their study approximately 6-years prior to ours. Our results were also consistent with a GC–MS-based metabolomics study that did not find significantly different metabolite changes in IC/BPS urine after FDR correction^18^.

There are several limitations to our study. First, the APF peptide was not observed in our non-targeted LC–MS-based peptidomics analysis, and follow-up experiments revealed that the APF peptide was removed during the two-stage urinary peptide enrichment protocol (Fig. S8). Other peptides were also likely removed during sample preparation, and some of these undetected peptides may serve to differentiate IC/BPS from healthy controls. Thus, alternative sample preparation protocols to enrich for a different subset of analytes may benefit future peptidomics studies and the identification of other potential peptide biomarkers. Another important consideration is that our IC/BPS cohort was comprised exclusively of female patients to minimize any gender-dependent variability. Although IC/BPS affects predominantly women, men are also susceptible to IC/BPS and to the related syndrome CP/CPPS^2,9,10^. Future studies evaluating peptide profiles of urine from male IC/BPS and/or CP/CPPS patients will be important to ascertain if these findings are generally applicable or are specific to female IC/BPS patients. In addition, it will be of great importance to compare urinary peptide content of IC/BPS to other urological disorders to determine the usefulness of these peptides to discriminate IC/BPS from other disorders with similar symptoms. Regardless, our study is a critical first step to examining the small peptide content of IC/BPS patient urine that paves the way for these future studies.

In summary, our study presents two major findings that will be beneficial for IC/BPS researchers in future. First, our study revealed differences in the profiles of small urinary peptides for IC/BPS patients compared to age-matched controls. Our results were consistent with increased protease activity in IC/BPS, although alternative explanations for these changes are possible. Second, our study enabled the direct measurement of APF peptide abundance in IC/BPS and control urine. Our results indicate that the full-length APF peptide was not consistently found in the urine of IC/BPS patients at levels sufficient to reliably differentiate IC/BPS patients from healthy individuals. As a result, future efforts to evaluate the APF peptide as a biomarker for IC/BPS may benefit from considering alternative forms of the APF peptide or by combining the APF peptide measurement data with additional putative biomarkers. Overall, our results may aid researchers in understanding the etiology of IC/BPS, and peptides identified here may serve as putative biomarkers to inform future diagnostic efforts.

## Supplementary Information

Supplementary Information 1.

Supplementary Information 2.

## Data Availability

The mass spectrometry peptidomics data have been deposited to the ProteomeXchange Consortium via the PRIDE^68^ partner repository with the dataset identifier PXD031843 and 10.6019/PXD031843.
