# Supplementary material for: Peptidomics analysis reveals changes in small urinary peptides in patients with interstitial cystitis/bladder pain syndrome
Source: Sci Rep. 2022 May 18;12:8289. doi: 10.1038/s41598-022-12197-2 (PMC9117215; doi:10.1038/s41598-022-12197-2)
Supplement: Supplementary file 2 — Supplementary Information 2. [file 41598_2022_12197_MOESM2_ESM.docx]

**Supporting Information**

**Peptidomics analysis reveals changes in small urinary peptides in patients with interstitial cystitis/bladder pain syndrome**

Md Shadman Ridwan Abid,^1‡^ Haowen Qiu,^2,3‡^ Bridget A. Tripp,^3^ Aline de Lima Leite,^3^ Heidi E. Roth,^1^ Jiri Adamec,^4,5^ Robert Powers,^1,3,5^* and James W. Checco^1,3^*

*^1^Department of Chemistry, University of Nebraska-Lincoln, Lincoln, NE, United States*

*^2^Center for Biotechnology, University of Nebraska-Lincoln, Lincoln, NE, United States*

*^3^The Nebraska Center for Integrated Biomolecular Communication (NCIBC), University of Nebraska-Lincoln, Lincoln, NE, United States*

*^4^Department of Biochemistry, University of Nebraska-Lincoln, Lincoln, NE, United States*

*^5^Redox Biology Center, University of Nebraska-Lincoln, Lincoln, NE, United States*

^‡^Denotes equal contribution

*corresponding authors: rpowers3@unl.edu; checco@unl.edu

**Table of contents**

Supporting Materials and MethodsS-3

Supporting Tables and FiguresS-5

Table S1. Patient demographics and symptom scoresS-5

Figure S1. Apparent protein concentration for non-targeted peptidomics samples S-6

Figure S2. Processing and normalization of peptidomics data S-7

Figure S3. Heat map of changing peptides S-8

Figure S4. MS/MS spectra of key peptides S-9

Figure S5. Individual peptide ROC curves S-18

Figure S6. APF peptide MS/MS data S-19

Figure S7. APF peptide MRM channels S-20

Figure S8. APF peptide recovery experiment S-21

Figure S9. Representative MRM chromatograms for urine samples S-22

Figure S10. APF peptide peak area versus GUPI scoreS-23

Figure S11. Protein concentration versus APF peptide detection S-24

Figure S12. PCA of LC-MS and NMR metabolomics analysis S-25

Supporting ReferencesS-26

**Supporting Materials and Methods**

***LC-MS-based metabolomics analysis.***

Urine samples were thawed and 40 µL were transferred to new microtubes containing cold methanol and internal standards. Quality control samples (QC) were prepared by mixing 10 µL from each of the samples within each batch. A blank with MilliQ water was prepared to track signals derived from contaminants inherent from sample handling. 100% methanol containing one internal standard for positive mode and one for negative mode were added to all samples, QCs, and blanks. Samples were then centrifuged at 14,000 rpm for 20 minutes at 4°C to pellet any protein. The supernatant was collected in a new tube and methanol was evaporated by speed vacuum centrifugation (SpeedVac R Plus, Savant). After dry, samples were kept at -80° C until analysis of the metabolome.

Metabolomics was performed with an Acquity UPLC equipped with an HSS T3 column (C18 column, dimension 1.0 mm x 50 mm, 1.7 mm particle size, Waters Corp.). The temperature of the column and auto-sampler were maintained at 40 °C and 15 °C, respectively. Metabolites were separated with an elution system consisting of water with 0.1% formic acid (solvent A) and acetonitrile with 0.1% formic acid (solvent B), and an 8-minute gradient from 0.1% B to 80% at a flow rate of 100 µL/min. Mass spectrometry was conducted with an Xevo G2-XS QTOF (Waters Corp. Milford, MA, USA) and electrospray ionization in the positive ion mode. The optimized MS experimental parameters were: a capillary voltage of 3.0 kV, a source temperature of 120 °C, a sampling cone voltage of 40 kV, a cone gas flow of 50 L/h, a desolvation temperature of 400 °C, a flow rate of 650 L/h, a scan range of 50–1200 *m/z*, a data acquisition rate of 0.5 s, and an MSE acquisition mode. The lower collision energy was off, and the higher collision energy was ramped from 15 to 40 V. To ensure accuracy, the *m/z* values of all ions acquired in the QTOF/MS were real-time adjusted by LockSpray calibrant Leucine-enkephalin ([M+H]^+^ = 556.2771).

LC-MS raw data were imported, processed, normalized, and reviewed using Progenesis QI v.2.1 (Non-linear Dynamics, Newcastle, UK). Adducts of the same compound were automatically grouped during the deconvolution process using an adduct list ([M+H]^+^, [M+Na]^+^, [M+K]^+^, [M+NH_4_]^+^, [M+H–H_2_O]^+^, [M+H–2H_2_O]^+^, [M+ACN+H]^+^, [M+CH_3_OH+H]^+^ and [M+ACN+Na]^+^) pre-defined during raw data importing. All runs were automatically aligned against a QC reference. For peak picking, sensitivity, chromatographic peak width, and retention time limits were set to 3 min, 0.10 min, and default, respectively. A matrix containing 14277 features (compound ions) were generated and submitted for statistical analysis.

Following UPLC-QTOF/MS analysis, spectra of all samples were batch corrected using the statTarget*^1^* R package, and 8356 metabolite peak features were detected. To account for sample-to-sample variability, data were processed using rank invariant normalization and pareto scaling, implemented using R packages lumi and MetaboAnalyze, respectively.*^2-6^* Principle component analysis (PCA) was performed following normalization. There was no clear separation and considerable overlaps between the IC/BPS and control groups (Figure S12).

To identify metabolite peak features with differential abundance between IC/BPS and healthy control samples, univariate analysis including Benjamini-Hochberg (BH)-corrected t-test and Wilcoxon rank-sum tests were performed. There was no metabolite peak feature that passed both tests with p-value less than 0.05.

***NMR-based metabolomics analysis.***

Urine samples were thawed at 4 °C, combined with methanol at a 1:2 ratio, and then centrifuged at 14,000 rpm for 10 minutes at 4 °C. The supernatant was collected, snap frozen in liquid nitrogen, and freeze dried for 24 hours to remove methanol by speed vacuum centrifugation (SpeedVac R Plus, Savant), and water by lyophilization (FreeZone, Labconco). The samples were then stored at -80 °C until analysis of the metabolome.

Dried samples were reconstituted with 150 µL of a 50 mM phosphate buffer in 100% D_2_O at pH 7.2 (uncorrected) containing 50 µM of 3-(tetramethylsilane) propionic acid-2,2,3,3-D_4_ (TMSP) as a chemical shift standard. Samples were then centrifuged at 14,000 rpm for 20 minutes at 4 °C to remove any precipitant. All NMR experiments were collected at 298 K with a Bruker AVANCE III HD 700 MHz spectrometer equipped with a 5 mm quadruple resonance QCI-P cryoprobe (^1^H, ^13^C, ^15^N, and ^31^P) with z-axis gradients. A SampleJet automated sample changer with Bruker ICON-NMR software was used to collect all data. A 1D ^1^H NMR spectrum was collected for all samples. In addition, natural abundance 2D ^1^H-^13^C HSQC spectra were collected at 25% sparsity using a non-uniform sampling (NUS) schedule. 1D NMR spectra were processed using our MVAPACK software*^7^* (http://bionmr.unl.edu/mvapack.php) followed by machine learning with RStudio.*^8^*

Following NMR analysis, 235 metabolite features were detected. Data were normalized using rank invariant normalization and pareto scaling to account for sample-to-sample variability.*^2^* PCA was performed following normalization. There was no clear separation and considerable overlaps between the IC/BPS and control groups (Figure S12).

To identify metabolite features with differential abundance between IC/BPS and healthy control samples, univariate analysis including BH-corrected t-test and Wilcoxon rank-sum tests were performed. There was no feature that passed both tests with a p-value less than 0.05.

***APF peptide recovery tests.***

Synthetic APF peptide (Vivitide, CAR-24007-v) was spiked into 4 aliquots of commercial human female urine samples (BioIVT) to final concentrations of 0.2 μM, 0.4 μM, 0.6 μM or 0.8 μM. The solutions were acidified with 0.1% formic acid (final concentration). For each APF peptide concentration, 100 μL of the spiked urine sample was removed, corresponding to 20 pmol, 40 pmol, 60 pmol, or 80 pmol of the APF peptide, respectively, and processed as described in the main text using either the two-stage enrichment protocol (SPE and ProteoSpin) or only SPE. Following elution, samples were dried in a vacuum concentrator and stored at -20 °C until LC-MRM analysis. All dried samples were resuspended in 30 μL of water with 0.1% formic acid. 5 μL of each reconstituted sample was injected into the triple quadrupole mass spectrometer for analysis by LC-MRM. A representative example of the results obtained for the 0.8 μM APF peptide spiked sample is shown in Figure S8.

|  | **Non-targeted** | | |  | **Targeted** | | |
| --- | --- | --- | --- | --- | --- | --- | --- |
|  |  | **Control** | **IC/BPS** |  |  | **Control** | **IC/BPS** |
| General | n | 44 | 43 |  | n | 41 | 43 |
|  | %Female | 100 | 100 |  | %Female | 100 | 100 |
|  | Age (years) | 37.5 ± 12.7 | 36.0 ± 12.3 |  | Age (years) | 38.4 ± 12.3 | 39.1 ± 13.0 |
|  | Height (in) | 63.1 ± 9.9 | 63.1 ± 10.1 |  | Height (in) | 64.7 ± 2.3 | 63.4 ± 10.2 |
|  | Weight (lb) | 154.0 ± 30.1 | 140.8 ± 37.5 |  | Weight (lb) | 158.4 ± 32.3 | 144.4 ± 37.6 |
|  | BMI | 25.5 ± 6.8 | 23.7 ± 6.6 |  | BMI | 26.6 ± 5.2 | 24.1 ± 6.5 |
|  |  |  |  |  |  |  |  |
| Symptoms | GUPI score  (0-45) | 1.8 ± 2.7 | 31.1 ± 5.9 |  | GUPI score  (0-45) | 2.0 ± 2.9 | 30.7 ± 5.0 |
|  | Symptom duration (years) | N/A | 10.7 ± 10.0 |  | Symptom duration (years) | N/A | 11.2 ± 11.0 |
|  | Symptom questionnaire  Pain score  (1-10) | 0.0 ± 0.1 | 6.2 ± 1.8 |  | Symptom questionnaire  Pain score  (1-10) | 0.0 ± 0.0 | 6.0 ± 1.7 |
|  | Symptom questionnaire  Urgency score  (1-10) | 1.0 ± 1.4 | 6.1 ± 2.2 |  | Symptom questionnaire  Urgency score  (1-10) | 1.1 ± 1.6 | 6.2 ± 2.2 |
|  | Symptom questionnaire  Frequency score  (1-10) | 0.4 ± 0.8 | 5.6 ± 2.8 |  | Symptom questionnaire  Frequency score (1-10) | 0.5 ± 1.1 | 5.9 ± 2.4 |
|  |  |  |  |  |  |  |  |
| Reported race category | %White | 68.2 | 90.7 |  | %White | 61.0 | 90.7 |
|  | %Black | 9.1 | 2.3 |  | %Black | 19.5 | 0.0 |
|  | %American Indian | 0.0 | 0.0 |  | %American Indian | 0.0 | 0.0 |
|  | %Native Hawaiian | 0.0 | 0.0 |  | %Native Hawaiian | 0.0 | 0.0 |
|  | %Asian | 13.6 | 0.0 |  | %Asian | 14.6 | 4.7 |
|  | %Multirace | 2.3 | 2.3 |  | %Multirace | 2.4 | 2.3 |
|  | %Other | 4.5 | 4.7 |  | %Other | 0.0 | 2.3 |
|  | %Unknown | 2.3 | 0.0 |  | %Unknown | 2.4 | 0.0 |
|  |  |  |  |  |  |  |  |
| Reported ethnicity category | %Hispanic | 6.8 | 7.0 |  | %Hispanic | 2.4 | 7.0 |
|  | %Non-Hispanic | 93.2 | 93.0 |  | %Non-Hispanic | 97.6 | 93.0 |
|  | %Other | 0.0 | 0.0 |  | %Other | 0.0 | 0.0 |

**Table S1.** Patient demographics/reported survey responses for samples obtained from MAPP Research Network analyzed in both the non-targeted and targeted analysis. Values represent mean ± standard deviation.


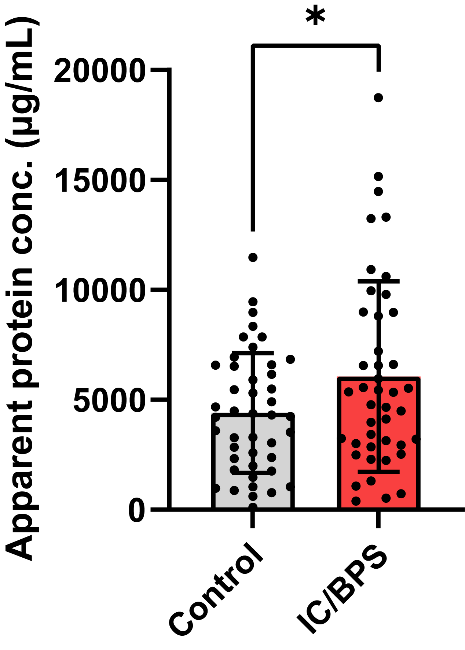


**Figure S1.** Apparent protein concentration (as determined by BCA) in the healthy control and IC/BPS patient urine samples used for the non-targeted peptidomics analysis. Apparent protein concentrations were determined after the C18 SPE enrichment step. Bars represented the mean ± standard deviation with individual values plotted as black circles. *p-value = 0.0353 (unpaired t-test).

**
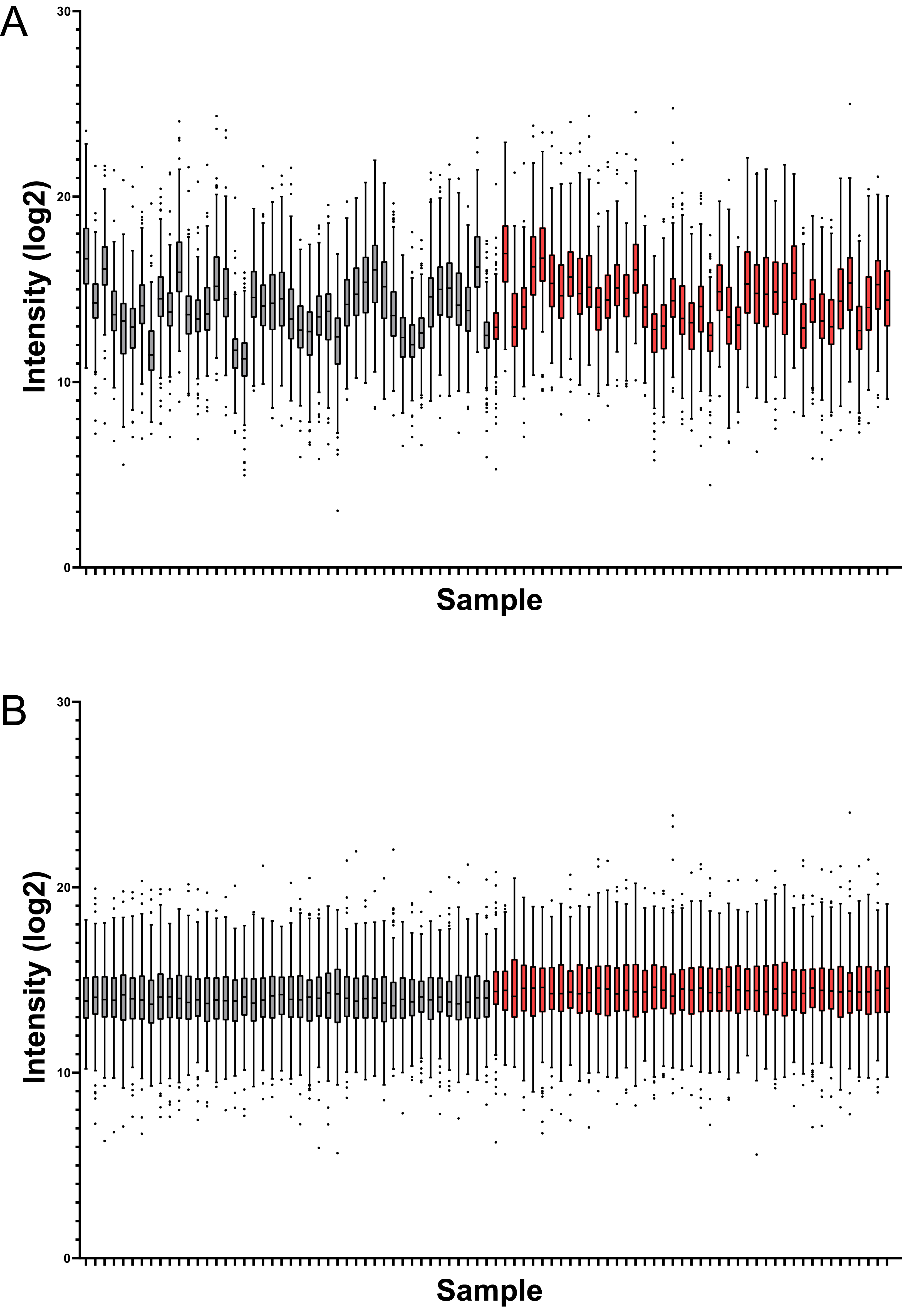
**

**Figure S2.** Processing and normalization of non-targeted peptidomics data. (A) Ion intensities across samples before normalization. (B) Ion intensities across samples after normalization. For both plots, middle lines indicate data set medians. Box extends from the 25^th^ to the 75^th^ percentiles and whiskers indicate 1.5 times the inter-quartile distance (Tukey method).


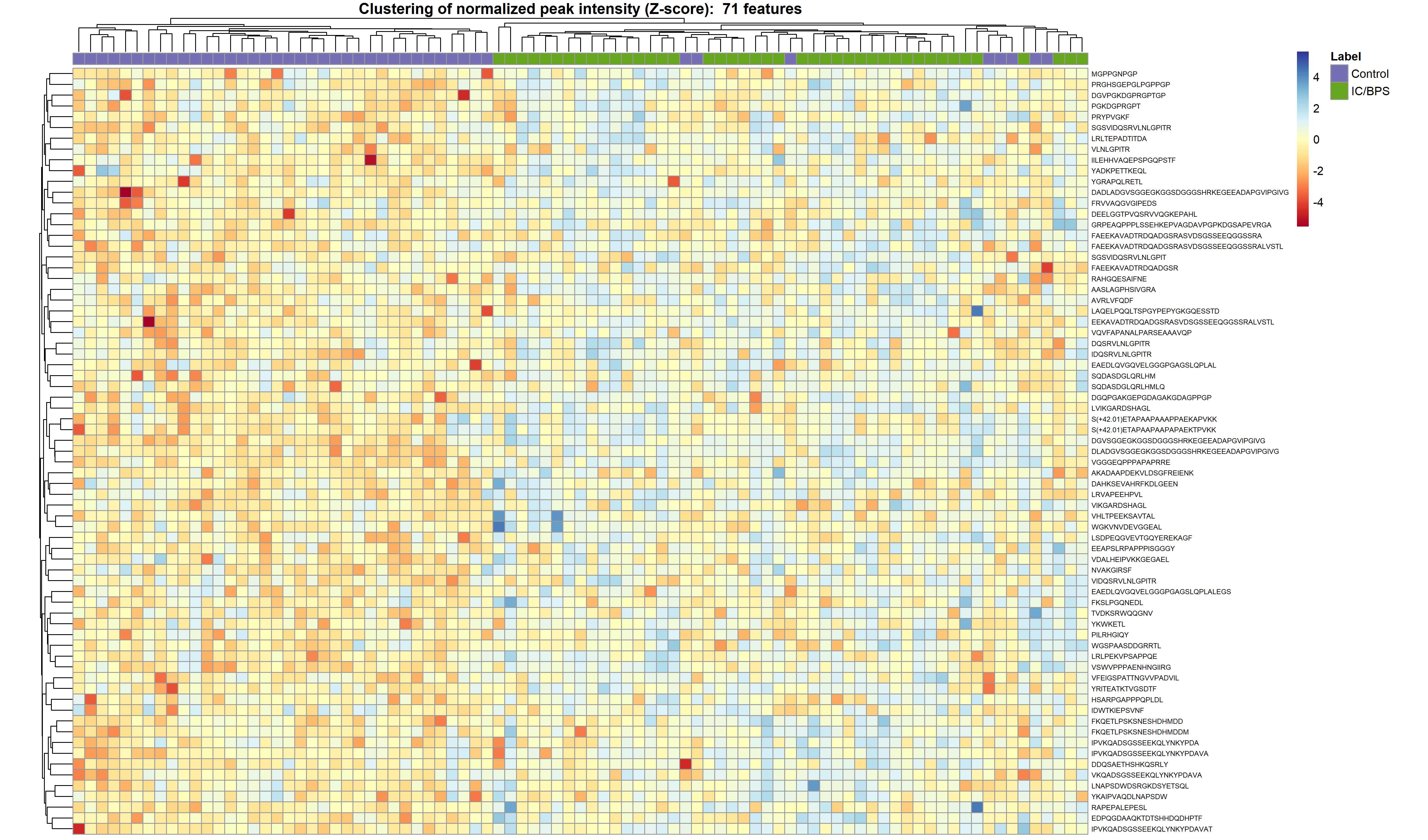


**Figure S3.** Heat map of significantly differentiated peptides. Columns represent individual urine samples, and the peptides are in the rows. Columns marked with a grey square indicate control samples, while columns marked with a green square indicate IC/BPS patient samples. The rows and columns were organized using hierarchical clustering.


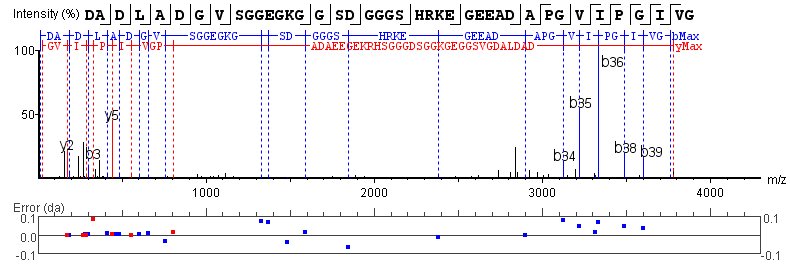


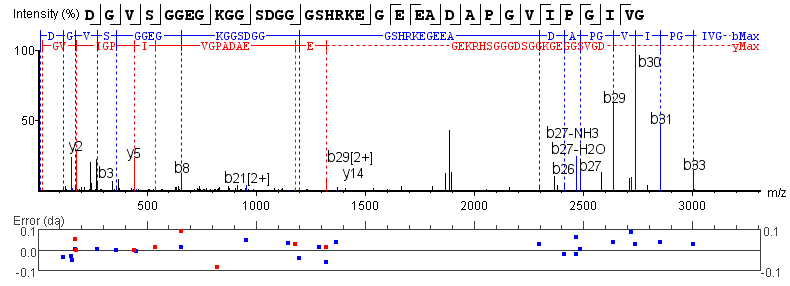


**Figure S4.** Representative annotated MS/MS spectra for the 18 peptides highlighted in Table 2 of the main text. Spectra were preprocessed and annotated using PEAKS Studio X software. Top line indicates peptide sequence and position of observed fragmentation generating b (left-pointing) and y (right-pointing) ions. Middle plot shows annotated MS/MS spectrum, highlighting b ions (blue) and y ions (red). Bottom plot shows mass error for each observed b ion (blue) and y ion (red). Figure continued on subsequent pages.


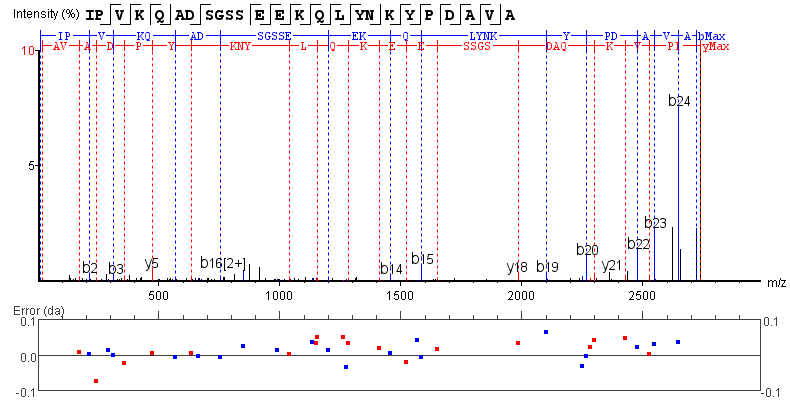


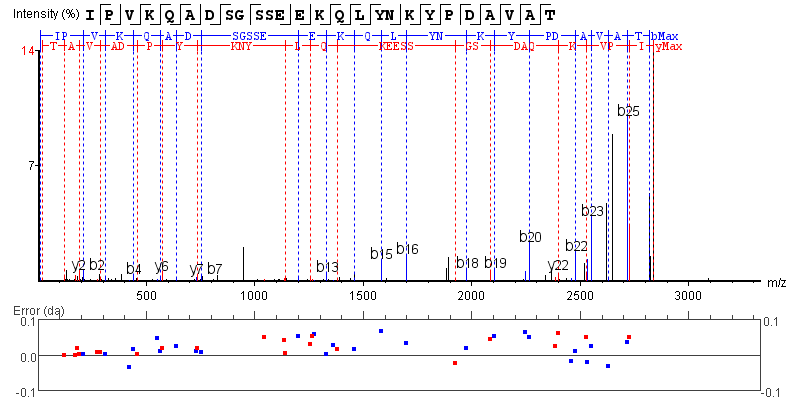


**Figure S4 (cont.).** Representative annotated MS/MS spectra for the 18 peptides highlighted in Table 2 of the main text. Spectra were preprocessed and annotated using PEAKS Studio X software. Top line indicates peptide sequence and position of observed fragmentation generating b (left-pointing) and y (right-pointing) ions. Middle plot shows annotated MS/MS spectrum, highlighting b ions (blue) and y ions (red). Bottom plot shows mass error for each observed b ion (blue) and y ion (red). Figure continued on subsequent pages.


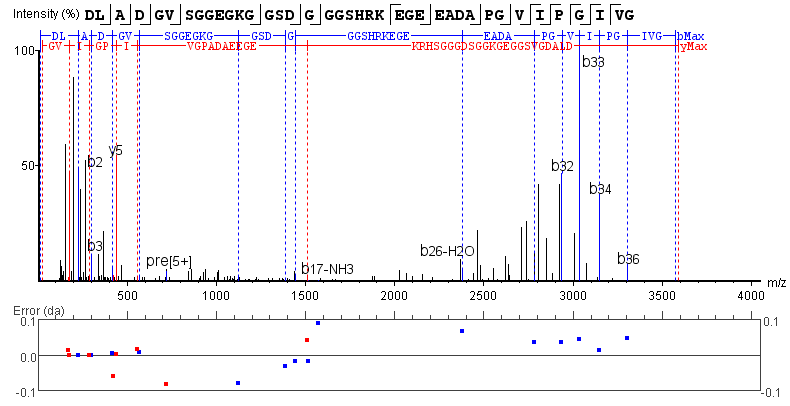


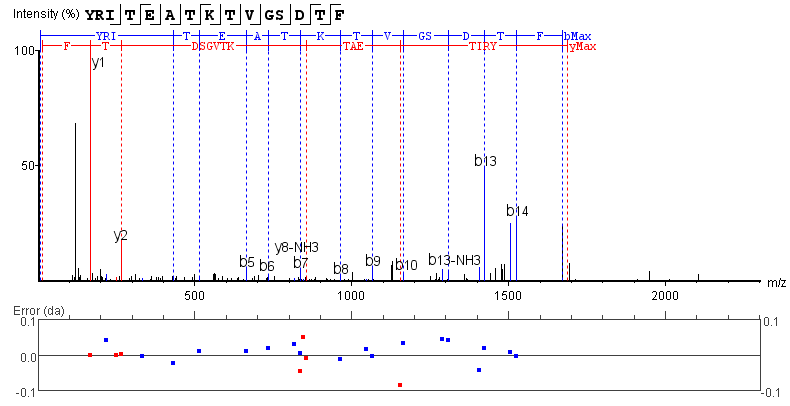


**Figure S4 (cont.).** Representative annotated MS/MS spectra for the 18 peptides highlighted in Table 2 of the main text. Spectra were preprocessed and annotated using PEAKS Studio X software. Top line indicates peptide sequence and position of observed fragmentation generating b (left-pointing) and y (right-pointing) ions. Middle plot shows annotated MS/MS spectrum, highlighting b ions (blue) and y ions (red). Bottom plot shows mass error for each observed b ion (blue) and y ion (red). Figure continued on subsequent pages.


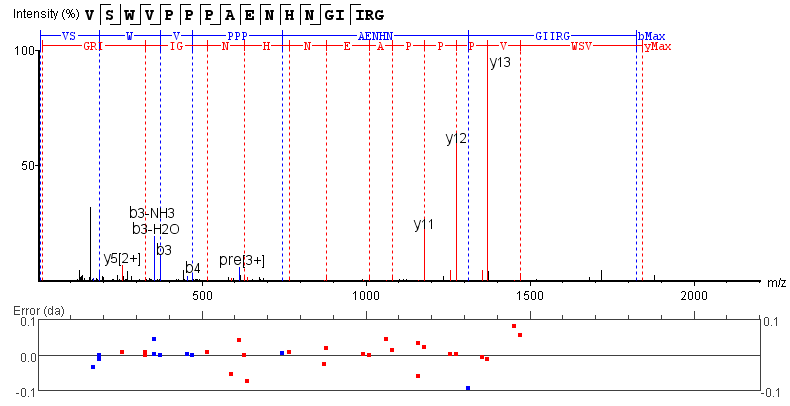


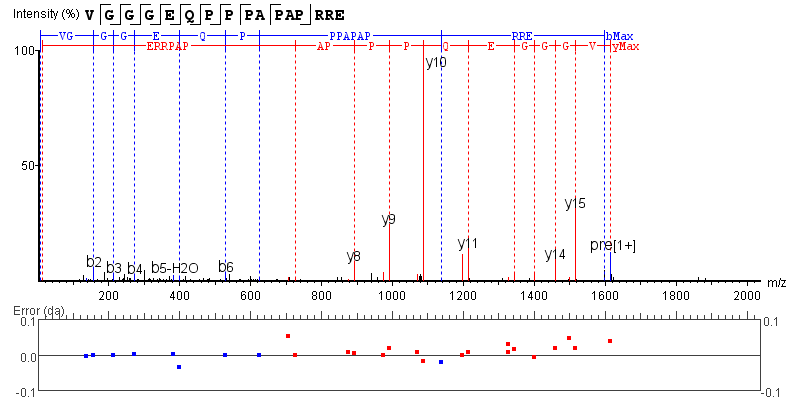


**Figure S4 (cont.).** Representative annotated MS/MS spectra for the 18 peptides highlighted in Table 2 of the main text. Spectra were preprocessed and annotated using PEAKS Studio X software. Top line indicates peptide sequence and position of observed fragmentation generating b (left-pointing) and y (right-pointing) ions. Middle plot shows annotated MS/MS spectrum, highlighting b ions (blue) and y ions (red). Bottom plot shows mass error for each observed b ion (blue) and y ion (red). Figure continued on subsequent pages.


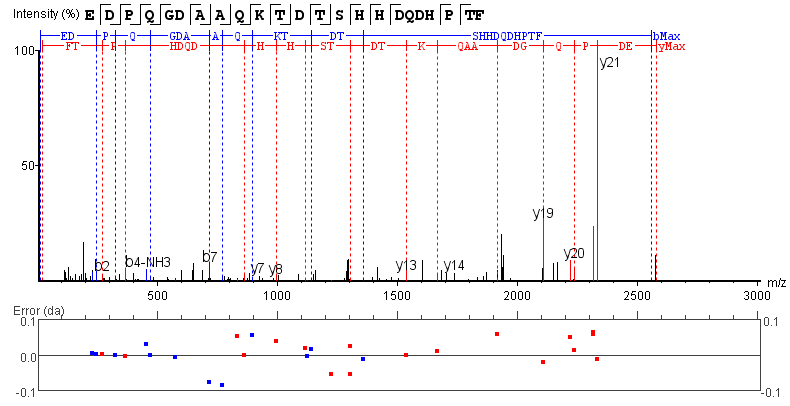


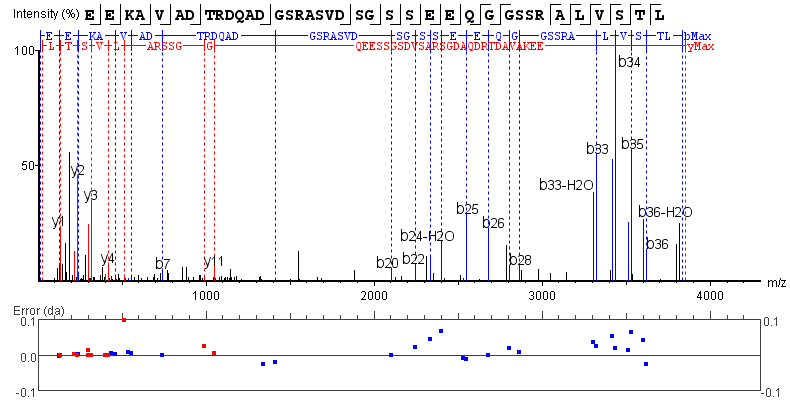


**Figure S4 (cont.).** Representative annotated MS/MS spectra for the 18 peptides highlighted in Table 2 of the main text. Spectra were preprocessed and annotated using PEAKS Studio X software. Top line indicates peptide sequence and position of observed fragmentation generating b (left-pointing) and y (right-pointing) ions. Middle plot shows annotated MS/MS spectrum, highlighting b ions (blue) and y ions (red). Bottom plot shows mass error for each observed b ion (blue) and y ion (red). Figure continued on subsequent pages.


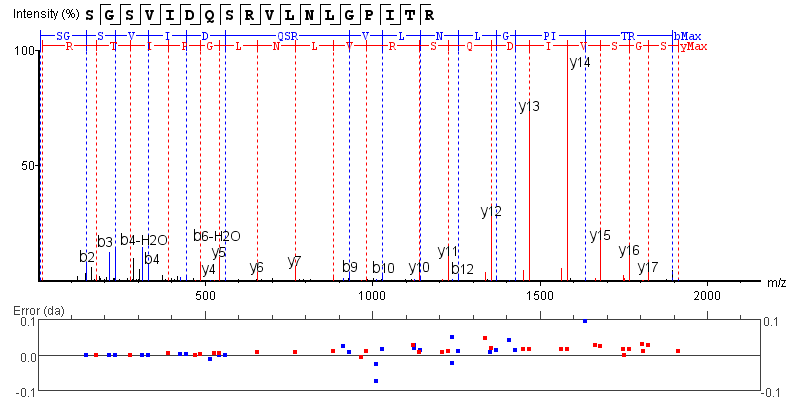


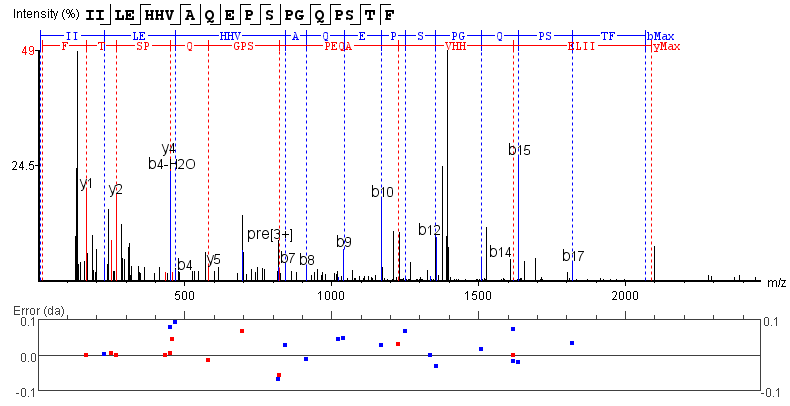


**Figure S4 (cont.).** Representative annotated MS/MS spectra for the 18 peptides highlighted in Table 2 of the main text. Spectra were preprocessed and annotated using PEAKS Studio X software. Top line indicates peptide sequence and position of observed fragmentation generating b (left-pointing) and y (right-pointing) ions. Middle plot shows annotated MS/MS spectrum, highlighting b ions (blue) and y ions (red). Bottom plot shows mass error for each observed b ion (blue) and y ion (red). Figure continued on subsequent pages.


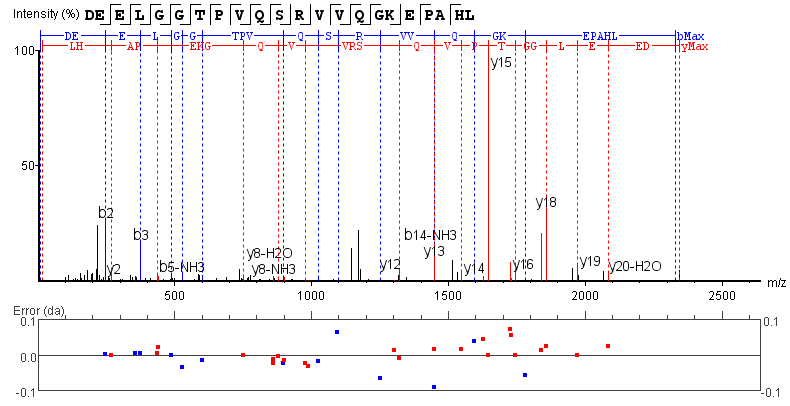


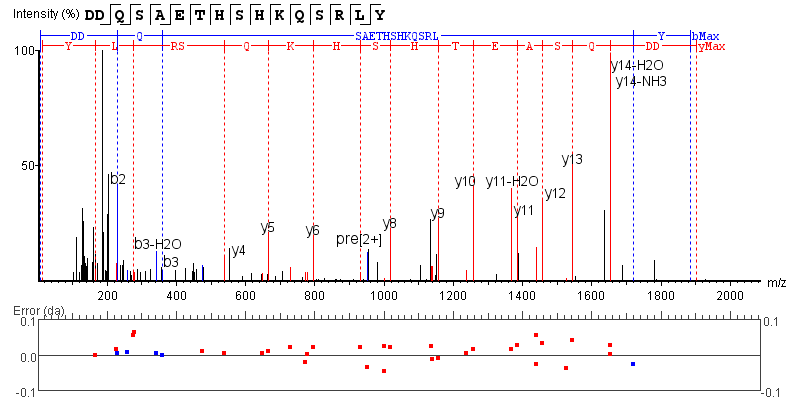


**Figure S4 (cont.).** Representative annotated MS/MS spectra for the 18 peptides highlighted in Table 2 of the main text. Spectra were preprocessed and annotated using PEAKS Studio X software. Top line indicates peptide sequence and position of observed fragmentation generating b (left-pointing) and y (right-pointing) ions. Middle plot shows annotated MS/MS spectrum, highlighting b ions (blue) and y ions (red). Bottom plot shows mass error for each observed b ion (blue) and y ion (red). Figure continued on subsequent pages.


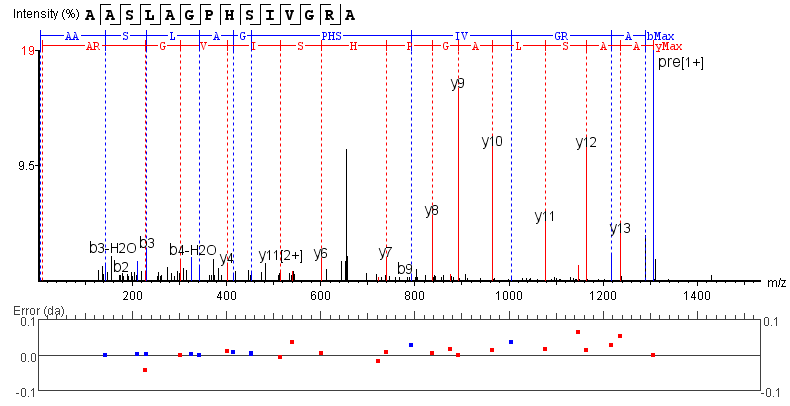


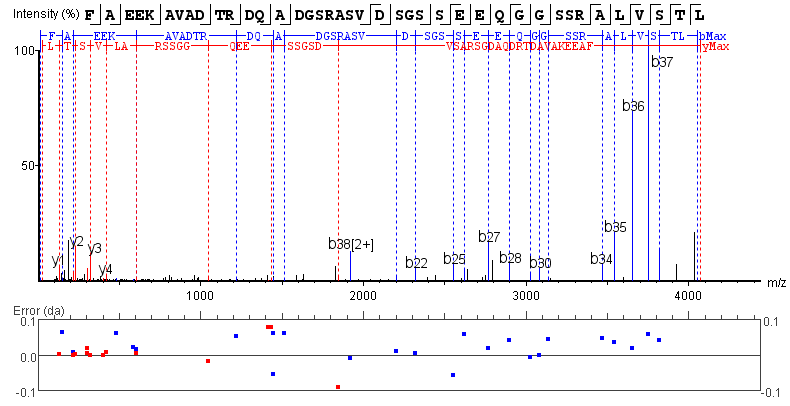


**Figure S4 (cont.).** Representative annotated MS/MS spectra for the 18 peptides highlighted in Table 2 of the main text. Spectra were preprocessed and annotated using PEAKS Studio X software. Top line indicates peptide sequence and position of observed fragmentation generating b (left-pointing) and y (right-pointing) ions. Middle plot shows annotated MS/MS spectrum, highlighting b ions (blue) and y ions (red). Bottom plot shows mass error for each observed b ion (blue) and y ion (red). Figure continued on subsequent pages.


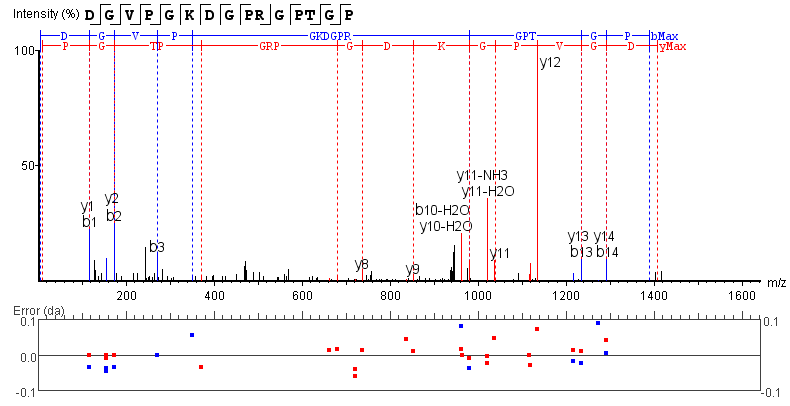

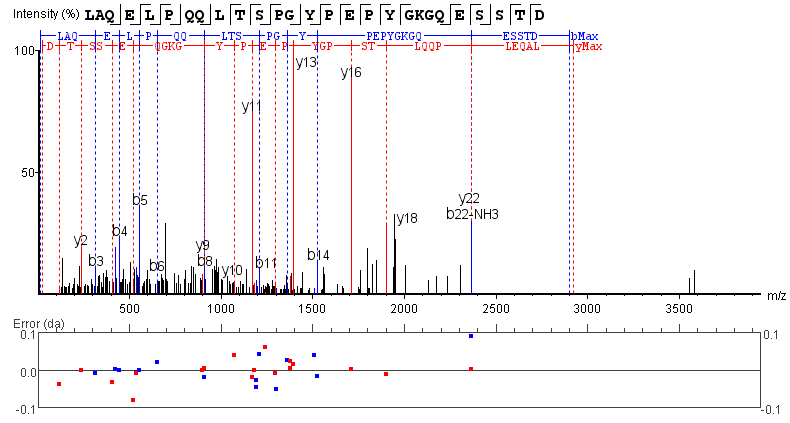


**Figure S4 (cont.).** Representative annotated MS/MS spectra for the 18 peptides highlighted in Table 2 of the main text. Spectra were preprocessed and annotated using PEAKS Studio X software. Top line indicates peptide sequence and position of observed fragmentation generating b (left-pointing) and y (right-pointing) ions. Middle plot shows annotated MS/MS spectrum, highlighting b ions (blue) and y ions (red). Bottom plot shows mass error for each observed b ion (blue) and y ion (red).

**
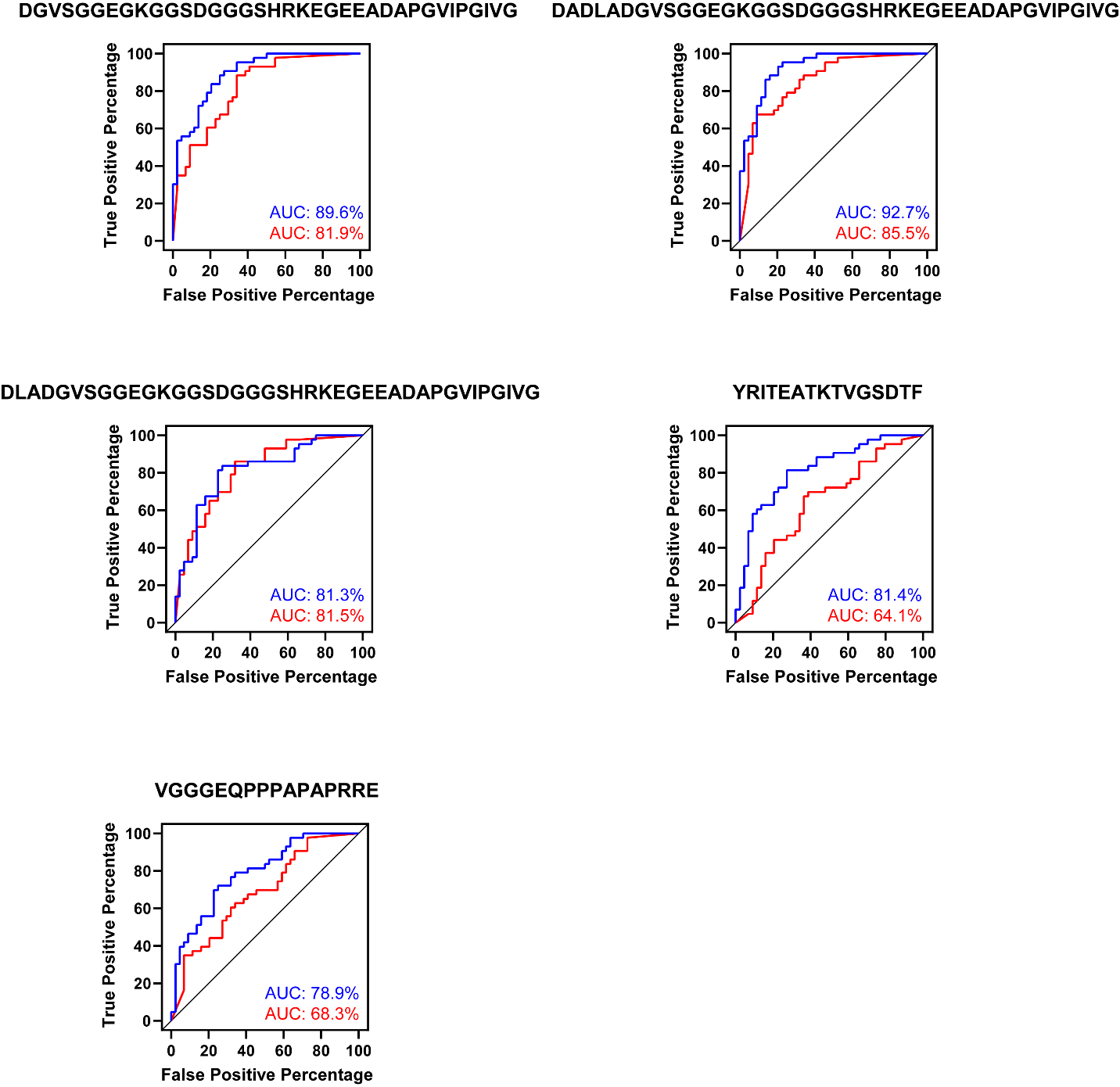
**

**Figure S5.** Individual receiver operating characteristic (ROC) curves for each of the five peptides with VIP scores > 2.00 from the PLS-DA model. ROC curves used either a logistic regression (blue) or random forest (red) model.

**
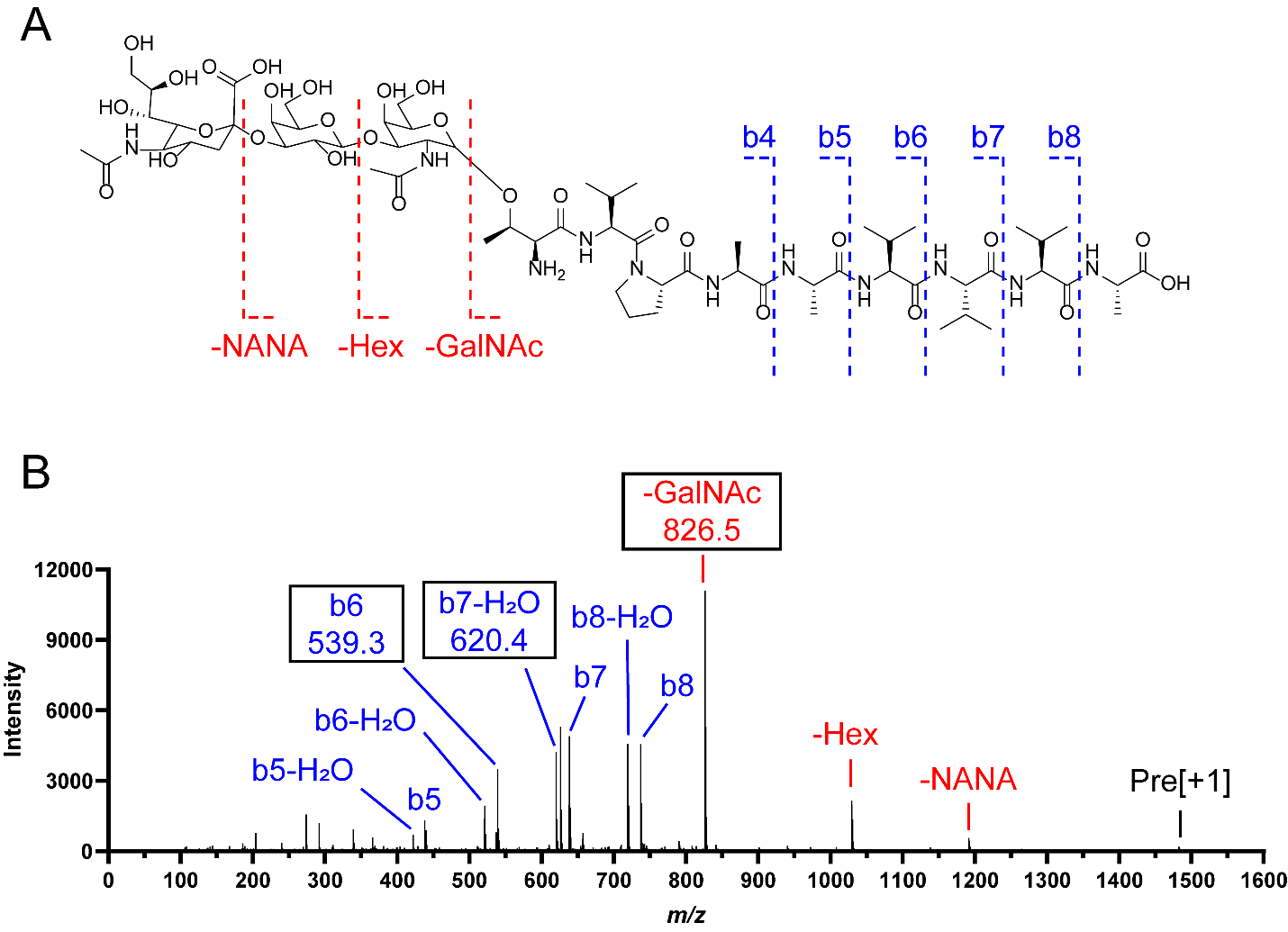
**

**Figure S6.** MS/MS analysis of the synthetic APF peptide. (A) Chemical structure of the APF peptide along with fragment annotations. (B) Annotated MS/MS spectrum (Q-TOF, CID) of the synthetic APF peptide. The ions in the black boxes with masses notated correspond to the representative MRM channels shown Figure S7. Note, the blue b ions were the result after complete loss of glycosylation. NANA = *N*-acetylneuraminic acid. Hex = Hexose. GalNAc = *N*-acetylgalactosamine.

**
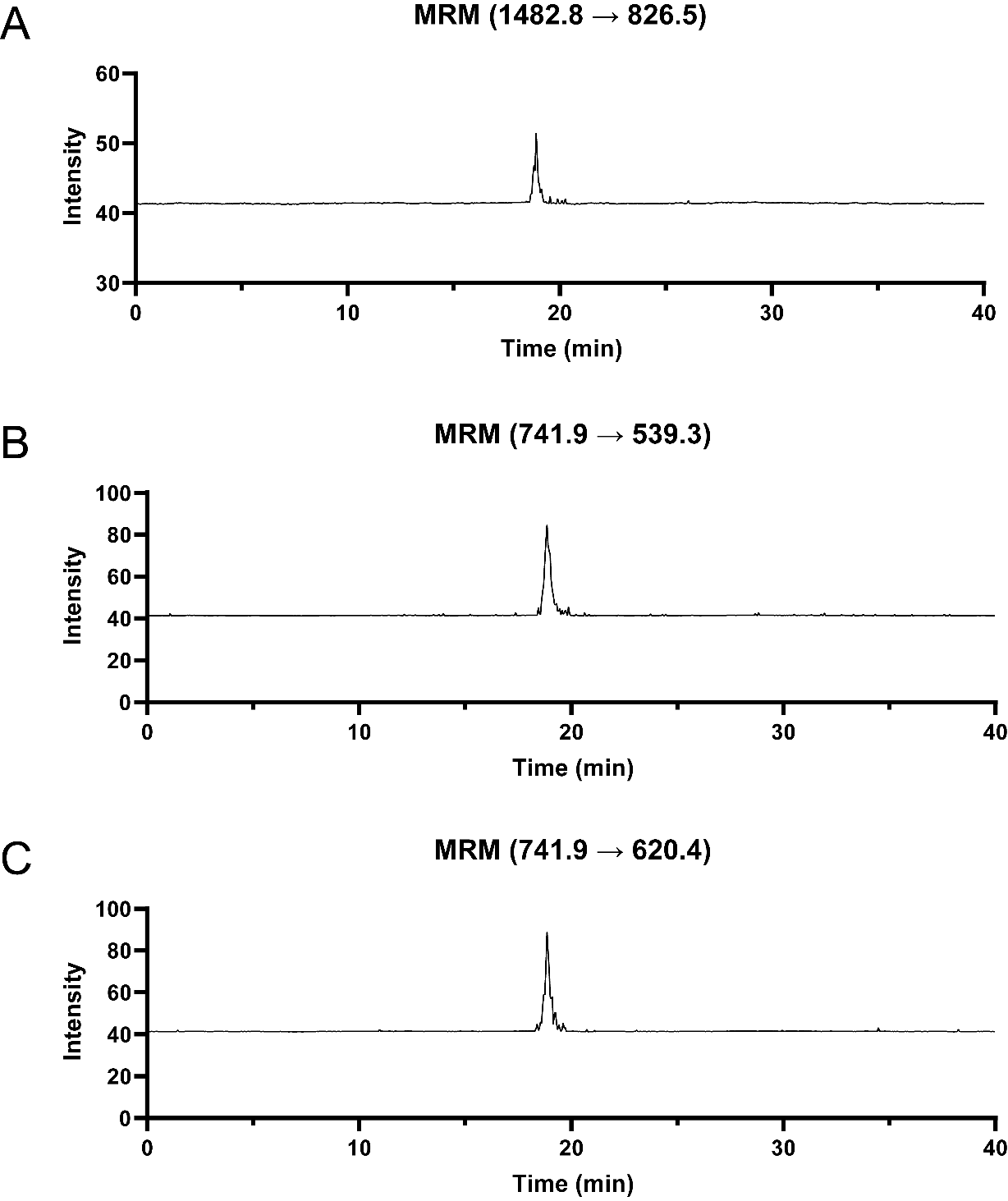
**

**Figure S7.** Representative LC-MRM chromatograms from an injection of the synthetic APF peptide. Each chromatogram shows one of three different transitions monitored for APF peptide detection. The graph titles indicate the precursor ion to fragment ion monitored for each LC-MRM channel.

**
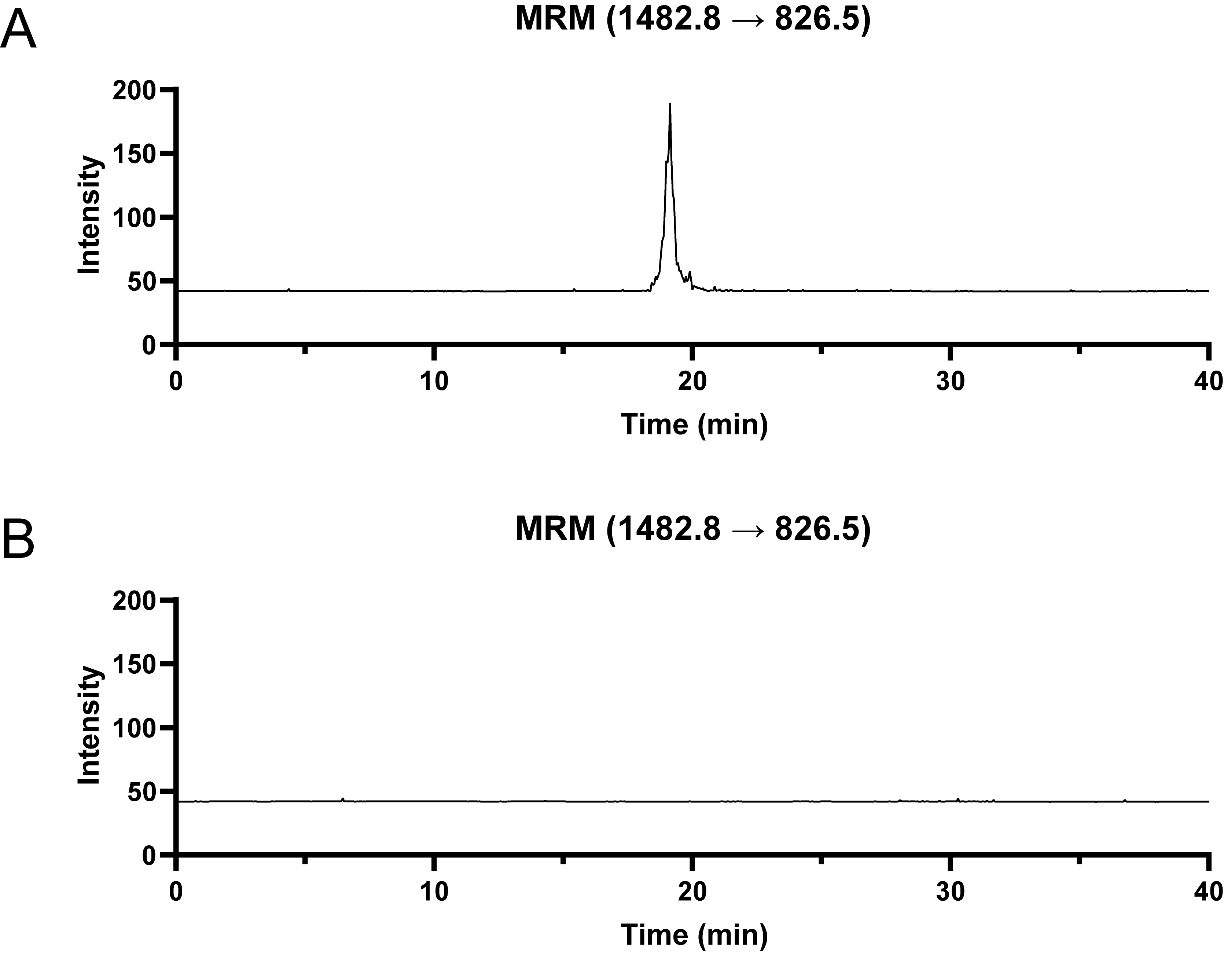
**

**Figure S8.** Representative LC-MRM chromatograms showing the recovery of 0.8 μM synthetic APF peptide spiked into 100 μL of commercial urine and processed by either (A) SPE only or (B) SPE followed by ProteoSpin. The graph titles indicate the precursor ion to fragment ion monitored for each LC-MRM channel.

**
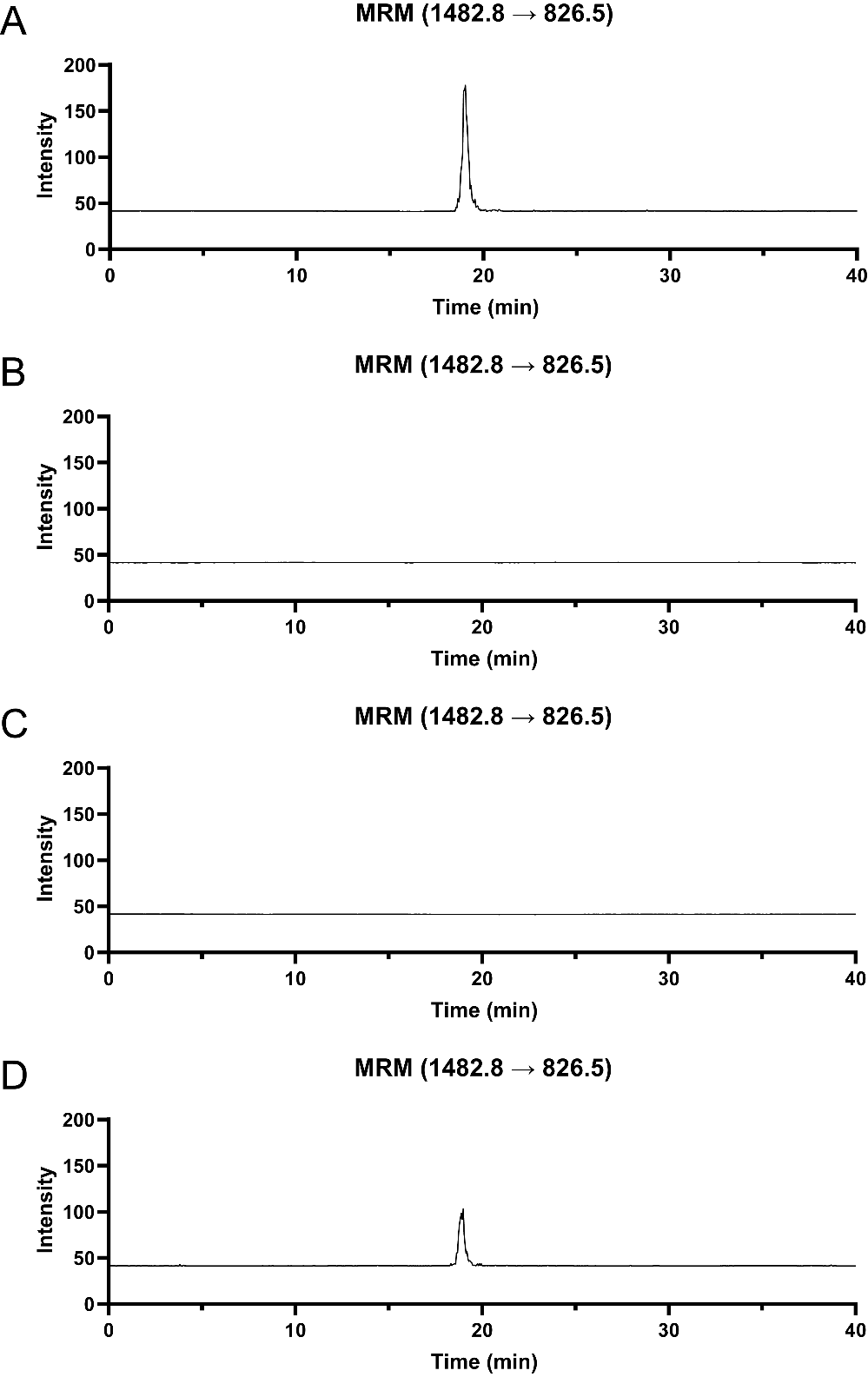
**

**Figure S9.** Representative LC-MRM chromatograms showing the presence or absence of the APF peptide in urine samples. (A) and (B) show two representative urine samples from IC/BPS patients. (C) and (D) show two representative urine samples from healthy controls. The graph titles indicate the precursor ion to fragment ion monitored for each LC-MRM channel.


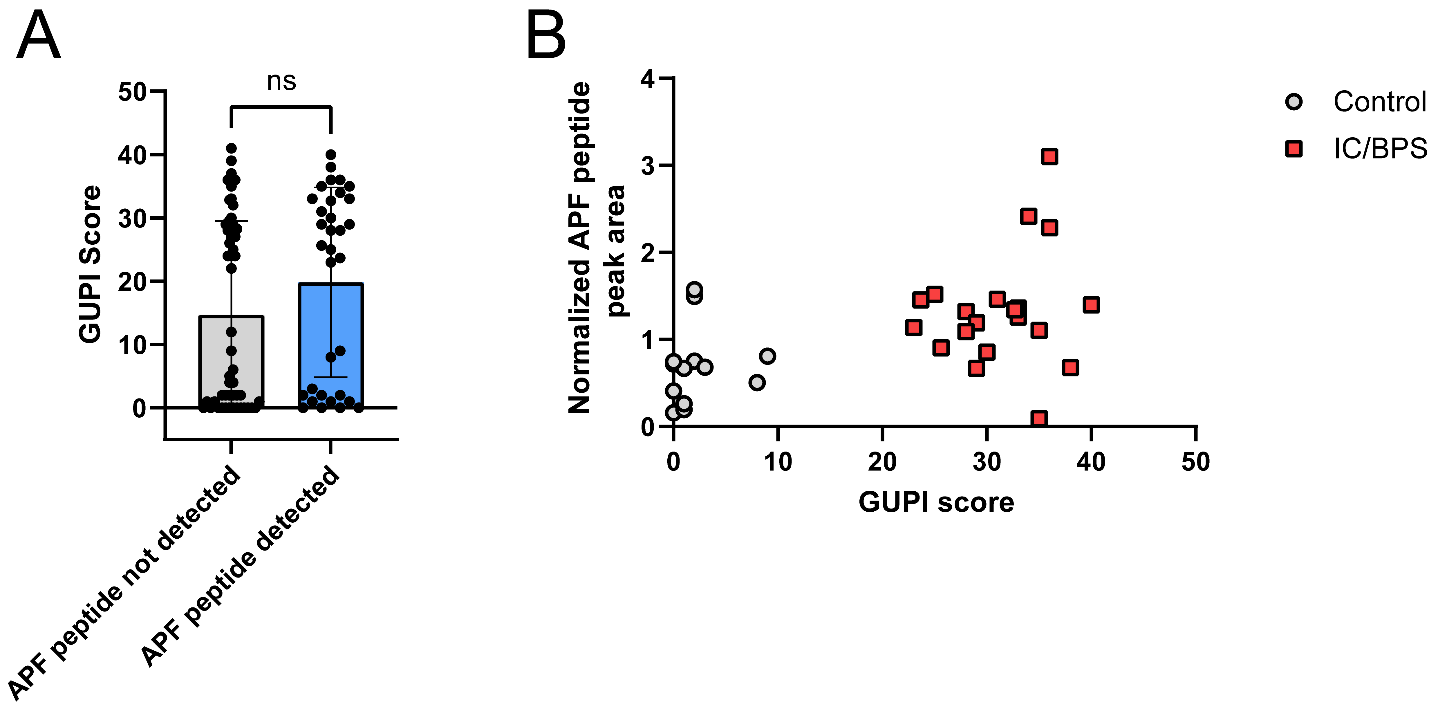


**Figure S10.** Relationship between APF peptide abundance and GUPI scores. (A) GUPI scores in samples with a detected APF peptide compared to samples without a detected APF peptide. Bars represented the mean ± standard deviation with individual values plotted as black circles. “ns” indicates a p-value > 0.05 (unpaired t-test). (B) Normalized APF peptide peak area as a function of GUPI score.


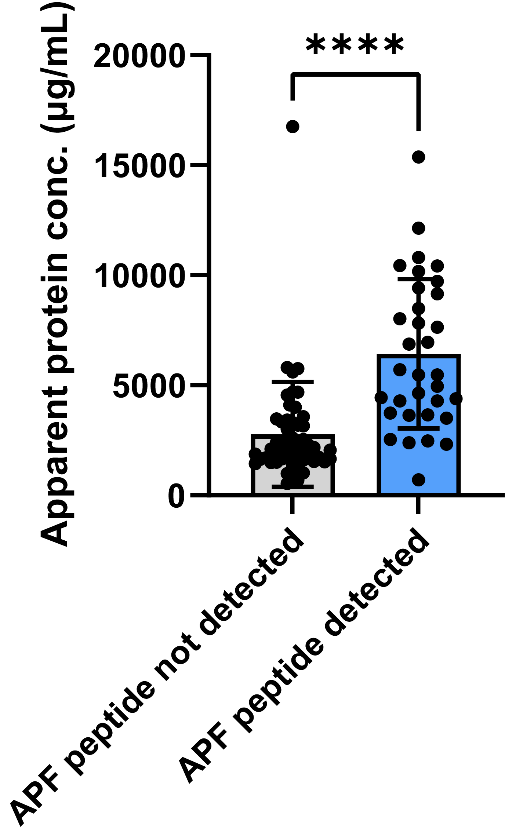


**Figure S11.** Apparent protein concentrations in samples with a detected APF peptide compared to samples without detected APF peptide. Apparent protein concentrations were determined by BCA assay after C18 SPE. Bars represented the mean ± standard deviation with individual values plotted as black circles. **** p-value < 0.0001 (unpaired t-test).

**
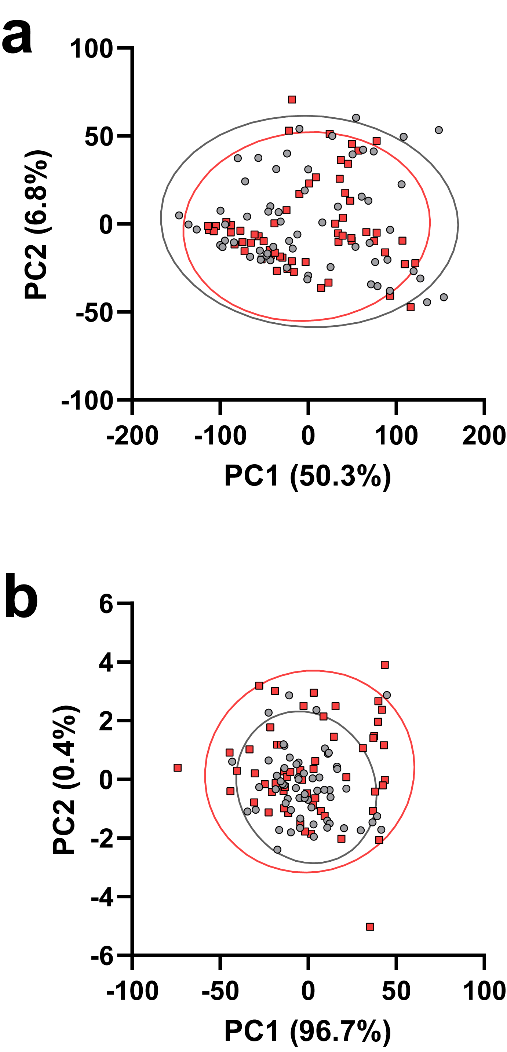
**

**Figure S12.** PCA scores plots for (a) LC-MS metabolomics analysis and (b) NMR metabolomics analysis, both after rank invariant normalization. For both analyses, no separation between IC/BPS patients (red) and healthy controls (grey) was observed.

**Supporting References**

1. Luan, H., Ji, F., Chen, Y., and Cai, Z. (2018) statTarget: A streamlined tool for signal drift correction and interpretations of quantitative mass spectrometry-based omics data, *Anal. Chim. Acta* *1036*, 66-72.

2. Du, P., Kibbe, W. A., and Lin, S. M. (2008) lumi: a pipeline for processing Illumina microarray, *Bioinformatics* *24*, 1547-1548.

3. Du, P., Kibbe, W. A., and Lin, S. M. (2007) nuID: a universal naming scheme of oligonucleotides for illumina, affymetrix, and other microarrays, *Biol Direct* *2*, 16.

4. Du, P., Zhang, X., Huang, C. C., Jafari, N., Kibbe, W. A., Hou, L., and Lin, S. M. (2010) Comparison of Beta-value and M-value methods for quantifying methylation levels by microarray analysis, *BMC Bioinf.* *11*, 587.

5. Lin, S. M., Du, P., Huber, W., and Kibbe, W. A. (2008) Model-based variance-stabilizing transformation for Illumina microarray data, *Nucleic Acids Res.* *36*, e11.

6. Gift, N., Gormley, I. C., and Brennan, L. (2010) MetabolAnalyze: probabilistic principal components analysis for metabolomic data, *R package version 1.3.1*.

7. Worley, B., and Powers, R. (2014) MVAPACK: a complete data handling package for NMR metabolomics, *ACS Chem. Biol.* *9*, 1138-1144.

8. RStudioTeam. (2021) RStudio: Integrated Development for R, Boston, MA.
